# Evaluation of a Web-Based Culturally Sensitive Educational Video to Facilitate Informed Cervical Cancer Screening Decisions Among Turkish- and Moroccan-Dutch Women Aged 30 to 60 Years: Randomized Intervention Study

**DOI:** 10.2196/35962

**Published:** 2022-10-26

**Authors:** Nora Hamdiui, Mart L Stein, Jim van Steenbergen, Rik Crutzen, Martine Bouman, Abresham Khan, Miyase N Çetin, Aura Timen, Maria van den Muijsenbergh

**Affiliations:** 1 National Coordination Centre for Communicable Disease Control Centre for Infectious Disease Control National Institute for Public Health and the Environment Bilthoven Netherlands; 2 Department of Primary and Community Care Radboud Institute for Health Sciences Radboud University Medical Center Nijmegen Netherlands; 3 Leiden University Medical Centre Centre for Infectious Diseases Leiden Netherlands; 4 Department of Health Promotion Care and Public Health Research Institute Maastricht University Maastricht Netherlands; 5 Center for Media & Health Gouda Netherlands; 6 Athena Institute for Research on Innovation and Communication in Health and Life Sciences Free University Amsterdam Amsterdam Netherlands; 7 Program Prevention and Care Pharos: Dutch Centre of Expertise on Health Disparities Utrecht Netherlands

**Keywords:** cervical cancer, screening, informed decision-making, web-based intervention, culturally sensitive educational video, Turkish, Moroccan, The Netherlands

## Abstract

**Background:**

In the Netherlands, since 1996, a national cervical cancer (CC) screening program has been implemented for women aged 30 to 60 years. Regional screening organizations send an invitation letter and information brochure in Dutch to the home addresses of targeted women every 5 years. Although this screening is free of charge, Turkish- and Moroccan-Dutch women, especially, show low screening participation and limited informed decision-making (IDM). As Turkish- and Moroccan-Dutch women indicated their need for information on the practical, emotional, cultural, and religious aspects of CC screening, we developed a culturally sensitive educational video (CSEV) as an addition to the current information brochure.

**Objective:**

In this study, we aimed to evaluate the added effect of the CSEV on IDM regarding CC screening participation among Turkish and Moroccan women aged 30 to 60 years in the Netherlands through a randomized intervention study.

**Methods:**

Initial respondents were recruited via several social media platforms and invited to complete a web-based questionnaire. Following respondent-driven sampling, respondents were asked to recruit a number of peers from their social networks to complete the same questionnaire. Respondents were randomly assigned to the control (current information brochure) or intervention condition (brochure and CSEV). We measured respondents’ knowledge and attitude regarding CC screening and their intention to participate in the next CC screening round before and after the control or intervention condition. We evaluated the added effect of the CSEV (above the brochure) on their knowledge, attitude, intention, and IDM using intention-to-treat analyses.

**Results:**

The final sample (n=1564) included 686 (43.86%) Turkish and 878 (56.14%) Moroccan-Dutch women. Of this sample, 50.7% (793/1564) were randomized to the control group (350/793, 44.1% Turkish and 443/793, 55.9% Moroccan) and 49.3% (771/1564) to the intervention group (336/771, 43.6% Turkish and 435/771, 56.4% Moroccan). Among the Turkish-Dutch women, 33.1% (116/350) of the control respondents and 40.5% (136/336) of the intervention respondents consulted the brochure (not statistically significant). Among Moroccan-Dutch women, these percentages were 28.2% (125/443) and 37.9% (165/435), respectively (*P*=.003). Of all intervention respondents, 96.1% (323/336; Turkish) and 84.4% (367/435; Moroccan) consulted the CSEV. The CSEV resulted in more positive screening attitudes among Moroccan-Dutch women than the brochure (323/435, 74.3% vs 303/443, 68.4%; *P*=.07). Women, who had never participated in CC screening before, showed significantly more often a positive attitude toward CC screening compared with the control group (*P*=.01).

**Conclusions:**

Our short and easily implementable CSEV resulted in more positive screening attitudes, especially in Moroccan-Dutch women. As the CSEV was also watched far more often than the current brochure was read, this intervention can contribute to better reach and more informed CC screening decisions among Turkish- and Moroccan-Dutch women.

**Trial Registration:**

International Clinical Trial Registry Platform NL8453; https://tinyurl.com/2dvbjxvc

## Introduction

### Background

Cervical cancer (CC) is ranked as the fourth most frequently diagnosed cancer in women worldwide [1]. Since the introduction of widespread screening programs, there has been a decline in early- and late-stage CC [2].

In The Netherlands, since 1996, a national CC screening program has been implemented for women aged 30 to 60 years. Regional screening organizations send an invitation letter and information brochure in Dutch to the home addresses of targeted women every 5 years. Screening is free of charge and is carried out by the general practitioner (GP) or their practice assistant who samples a cervical smear (ie, clinician-based sampling). The smear is initially tested for the presence of high-risk human papillomavirus (hrHPV), a risk factor for developing CC [3]. If hrHPV is present, the cervical cells in the smear are assessed for abnormal or precancerous lesions. An important advantage of HPV-based screening is that it can also be performed by self-sampling. If this self-sample tests positive for hrHPV, a cervical smear for cytological examination is sampled at the GP’s office.

From an individual’s perspective, deciding to participate in screening involves careful consideration of the uncertain benefits and risks of adverse effects. This consideration is pivotal in informed decision-making (IDM), the process in which individuals base their decisions by optimal use of the information and weighing all the aspects involved. IDM is only possible when a woman has adequate decision-relevant knowledge and her attitude toward participating is consistent with her (intended) participation [4].

In the Netherlands, especially Turkish- and Moroccan-Dutch women, representing the largest immigrant population, show low screening participation and limited IDM regarding participation [5,6]. Earlier research indicated an overall lack of knowledge and nonfamiliarity with the possible disadvantages of CC screening [5].

In decision-making, Turkish- and Moroccan-Dutch women consider not only factual medical information but also practical, emotional, cultural, and religious aspects before deciding whether to screen for CC [5]. However, the current invitation letters and information brochures predominantly contain factual medical information. Turkish and Moroccan-Dutch women often indicated not (thoroughly) reading the invitation letter and brochure, or simply being unable to understand these materials due to a lack of good command of the Dutch language [5]. These women were also shown to make less use of printed media and more of audiovisual media [7]. As a culturally competent educational film, which was developed with peer educators, was successful in improving IDM for prenatal screening among pregnant ethnic minority women, we considered this beneficial for IDM in CC screening participation [8]. Thus, we developed a culturally sensitive educational video (CSEV) that incorporates more affective information and distributed it via respondent-driven sampling (RDS).

### Objectives

In this study, we evaluated the effect of the CSEV on IDM regarding CC screening participation among Turkish- and Moroccan-Dutch women. We hypothesized that adding a CSEV to the current Dutch information brochure would increase the IDM to participate in CC screening among these women.

## Methods

### Study Design

Between November 23, 2020, and August 6, 2021, a randomized intervention study was conducted with control and intervention groups. We used web-based RDS to recruit Turkish- and Moroccan-Dutch women, as previous attempts have shown that traditional random sampling methods are not effective in reaching these populations effectively [9]. Their close-knit social networks also enable respondents to recruit each other easily [10]. The reporting of this study adheres to the CONSORT (Consolidated Standards of Reporting Trials) guidelines.

### Randomization and Masking

Respondents were asked to complete a web-based questionnaire, in which questions on IDM were asked before and after the control or intervention condition. The control group was asked to read the information brochure regarding the screening program that is currently sent with the screening invitation. The intervention group was asked to read the same brochure and watch the CSEV. This request was displayed on a web page. By clicking *Next*, they first received the brochure, and subsequently on the next page, the CSEV was displayed.

**Figure 1:**
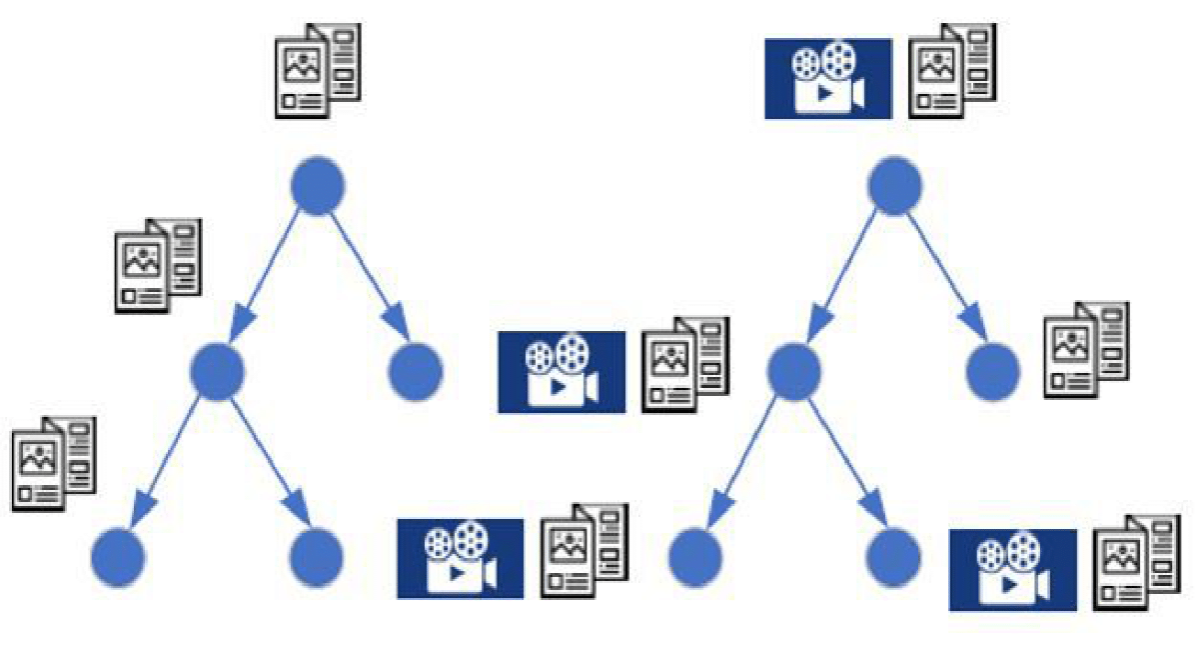
Study design: respondent-driven sampling where each new respondent was randomly assigned to either the control or intervention group.

RDS starts with a convenient, ideally diverse, sample of members of the population called seeds [11]. Seeds were asked to complete a questionnaire and recruit a number of their peers to complete the same questionnaire. The successfully recruited peers were then also asked to recruit a number of peers. This recruitment process was continued until the calculated sample size was reached. Unique tokens were used to follow who recruited whom and draw recruitment trees. Each new respondent was randomly assigned to either the control or the intervention condition (ie, individual-level randomization; Figure 1).

### Study Population and Recruitment

The inclusion criteria for respondents were as follows: the women must be (1) aged 30 to 60 years, (2) born in Turkey or Morocco and have at least one parent born in Turkey or Morocco (first-generation immigrants) or born in the Netherlands and have at least one parent born in Turkey or Morocco (second-generation immigrants), and (3) living in the Netherlands.

Seeds were recruited via several social media platforms, such as (1) public and private women’s groups on Facebook, (2) LinkedIn pages of the involved researchers, (3) the foundation called the Association Moroccan Doctors Netherlands, (4) the participating video producer Zouka Media, and (5) Instagram, wherein we contacted several influencers with many Turkish- and Moroccan-Dutch female followers and asked them to share the questionnaire via their story or bio. Throughout the study, we used paper- and web-based flyers and web-based infographics to promote and share the link to the questionnaire. The flyers and infographics were spread among offline community organizations, foundations, and mosques, as well as web-based platforms, such as LinkedIn and Facebook.

After completion of the questionnaire, respondents were asked to invite—through WhatsApp, platforms such as Instagram, and/or SMS text messaging—a maximum of 20 women from their social network to complete the same questionnaire. Via email, reminders were sent to complete and/or forward the questionnaire and to encourage respondents to remind their peers to complete the questionnaire (after 1 week of no participation of at least one peer). To prevent respondents from potentially influencing each other’s answers, respondents were explicitly requested not to discuss their answers or watch the CSEV with others. Initially, an incentive of €10 (US $9.69) was awarded to every respondent who completed the questionnaire herself and peer recruited 2 other women who also completed the questionnaire. From March 3, 2021, to further stimulate peer recruitment, an incentive of €15 (US $14.53) was awarded to every respondent who completed the questionnaire herself and peer recruited 1 other woman who also completed the questionnaire.

### The Questionnaire

We developed a questionnaire for measuring IDM based on the rational decision model, which supposes that decision-making is based on a proper understanding of the potential benefits and adverse effects of cancer screening (decision-relevant knowledge) in the context of personal situations and preferences (attitude) [12]. The questionnaire contained 52 questions regarding sociodemographic characteristics, previous CC screening participation, knowledge of CC screening, attitude toward CC screening, and intention to participate in the next CC screening round. The questionnaire (in Dutch) can be found in Multimedia Appendix 1. All questions were closed ended, except for the month and year of birth, the 4 digits of the postal code, and the size of their social network on the web. We asked questions on knowledge, attitude, and intention for clinician-based sampling, whereas for self-sampling, we included questions on awareness, perceptions, and intention. The rationale for this difference was that the self-sampling method was only introduced in 2017, which meant that not every woman was aware of its existence. Therefore, instead of assessing their knowledge and attitude, we questioned their awareness and perceptions of self-sampling. Knowledge of CC screening was measured using 3 questions about the subsequent steps following a test result and the possibility of false-positive test results, with scores ranging from 0 to 4. Attitude toward CC screening was measured using 10 questions, with scores ranging from 0 to 10. These scores were transformed to 0 to 100 scores to facilitate interpretation, following an earlier study by Korfage et al [13]. In agreement with van den Berg et al [14] and Korfage et al [13], we classified scores in the range of 45 to 55 as a neutral attitude. Scores <45 were classified as having a negative attitude, whereas scores >55 were classified as having a positive attitude. Intention was measured by asking the respondents whether they intended to participate in the next CC screening round. All questions regarding attitude and intention had 3 response options: “Yes,” “I do not know,” and “No.”

Following earlier research, we combined knowledge, attitude, and intention to calculate IDM (yes or no) [4,8]. An informed decision was defined as having adequate knowledge (total score≥3.0), either a positive attitude (total score>55.0) and a positive intention or a negative attitude (total score<45.0) and a negative intention. All other combinations were defined as an uninformed decision.

The questionnaire was made available in Dutch, Turkish, and Moroccan-Arabic languages. As first-generation Turkish- and Moroccan-Dutch immigrants have low reading abilities, audio recordings in Dutch, Turkish, Moroccan-Arabic, and Moroccan-Berber (a spoken language) languages were made available. To ensure understandability, the questionnaire was extensively pretested among 4 low-literate Turkish- and Moroccan-Dutch women. It took women approximately 15 minutes to complete the questionnaire.

### Culturally Sensitive Educational Videos

We developed 3 CSEVs in collaboration with the video producer and 8 Turkish- and Moroccan-Dutch peer educators and actresses. As all respondents received the brochure containing cognitive information on CC screening, we focused the video on affective information related to CC screening (ie, experiences and fears). Turkish- and Moroccan-Dutch women especially need information on the practical, emotional, cultural, and religious aspects of CC screening [5]. Therefore, the CSEVs emphasized on 3 themes regarding clinician-based sampling and ensured balanced content in terms of possible benefits and adverse effects. The themes included “more assurance regarding health and the ability to prevent treatment, surgery, or death, and because of this, being there for their children”; “according to the Islam, a woman should take good care of her health”; and “anxiety, shame, and privacy.” For self-sampling, 2 themes were included, namely “it is easy and not painful to perform self-sampling” and “trust in themselves to correctly perform self-sampling and trust in the test result.” The CSEV was available in Turkish, Moroccan-Arabic, and Moroccan-Berber (all with Dutch subtitles) languages. Moroccan-Dutch respondents could choose either a Moroccan-Arabic–spoken or Moroccan-Berber–spoken video.

To verify whether the CSEVs were understandable and culturally appropriate, discussions on the web were held among experts on language, communication, culture, and CC (screening). The CSEVs were also pilot-tested in a small sample of Turkish- and Moroccan-Dutch women to verify whether the feasibility, content, and layout matched their needs and requirements. Through automatic registration by the questionnaire software, we measured whether and how long the respondents consulted the brochure (in both the control and intervention groups) and whether the intervention group actually watched the CSEV.

All CSEVs are available on the official webpage of the Dutch National Institute for Public Health and the Environment [15]. Further details regarding the development and tailoring of the CSEVs are reported elsewhere [16].

### Sample Size Calculation

We used a 2-sided test and assumed a binomial distribution, 95% CI, 80% power, and an absolute change of 10% in IDM. Therefore, 776 Turkish- and 794 Moroccan-Dutch women (in total; both the control and intervention groups) were needed. This absolute change of 10% in IDM was based on a previously reported study using a developed CSEV and observing an increase of 11% in IDM regarding prenatal screening among pregnant ethnic minority women in the Netherlands [8].

### Statistical Analysis

The flow of respondents’ inclusion was visualized. Possible insincere respondents (ie, those that probably participated for incentives only) were excluded from the data and were not eligible for an incentive whenever one of the following criteria was met: (1) the respondent *and* her recruitee completed the questionnaire in <5 minutes or (2) the respondent *or* her recruitee completed the questionnaire in <5 minutes, *and* there was <5 minutes between the start of the 2 participations. Respondents who indicated no migration background, indicated a migration background other than Turkish or Moroccan, or did not indicate their country of birth and/or that of their parent or parents, and those aged <30 or >60 years were also excluded.

Descriptive statistics were used to provide an overview of the sample characteristics and the proportion of respondents who viewed the brochure and CSEV. To analyze the potential additional effect of the CSEV compared with that of the brochure only, we conducted intention-to-treat analyses [17]. We assessed the differences in knowledge (or awareness in the case of self-sampling), attitude (or perceptions in the case of self-sampling), intention, and IDM (only for clinician-based sampling) between the control and intervention groups after the control or intervention condition using chi-square tests or Fisher exact tests.

As a post hoc analysis, we explored the open-field comments stated by the respondents at the end of our questionnaire to explain the differences found between Turkish- and Moroccan-Dutch women. A 2-sided *P* value of <.05 was considered statistically significant. All analyses were performed using the statistical software R (R Foundation for Statistical Computing; version 4.0.2).

### Ethics Approval and Consent to Participate

After the Medical Ethics Review Committee of the University Medical Centre Utrecht confirmed that the Medical Research Involving Human Subjects Act does not apply to this study (nr: 20/105), we registered the trial at the International Clinical Trial Registry Platform (trial ID: NL8453). Respondents were informed about the study (but did not know that there was a control group and an intervention group) and were asked to give their digital informed consent.

## Results

### Flow of the Inclusion of Respondents

**Figure 2:**
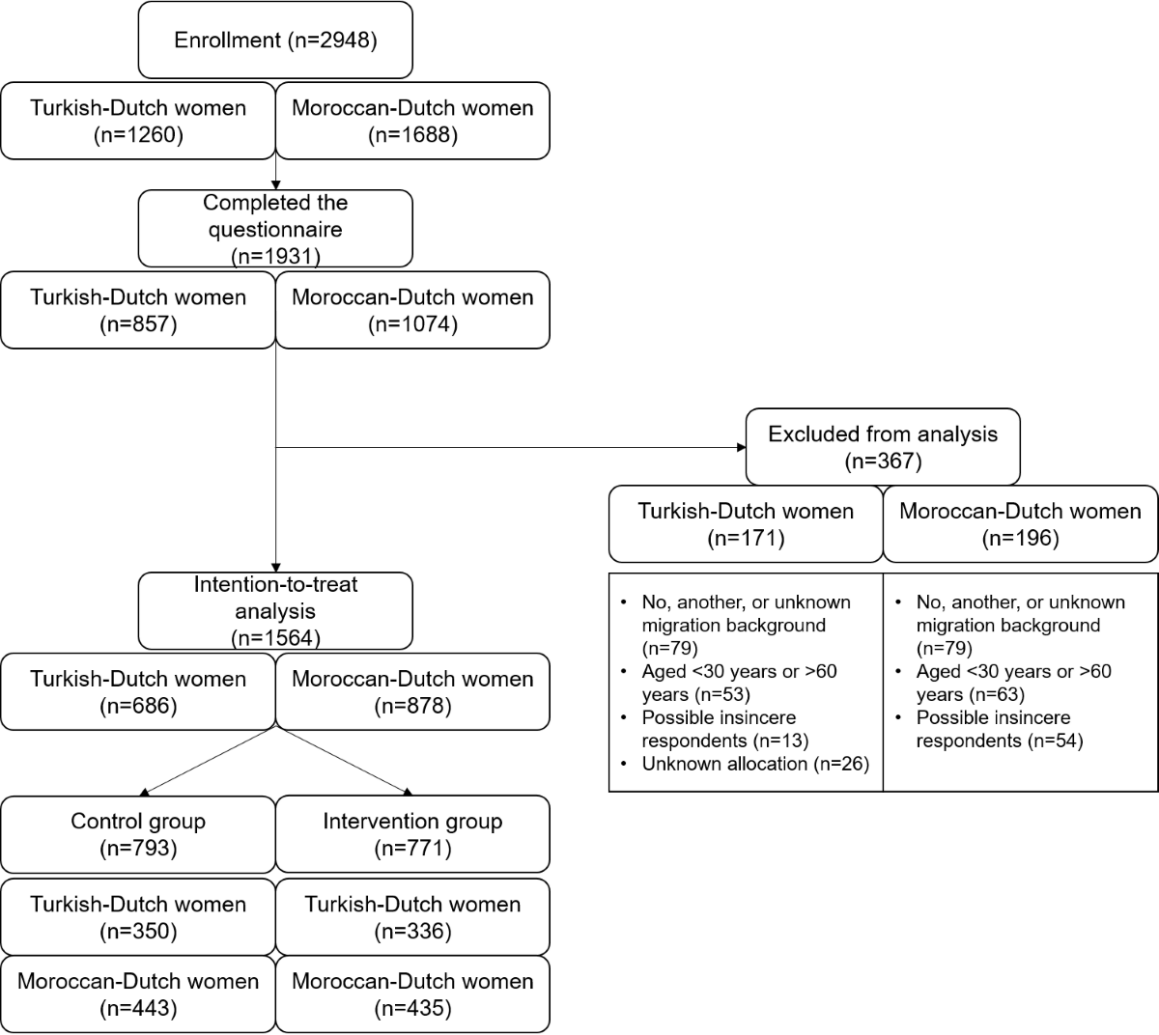
Flow diagram of the recruitment and response of respondents.

Of the 2948 respondents that started the questionnaire, 1931 (65.5%) completed it. After excluding 367 (19.01%) respondents, 1564 (80.99%) respondents were included in the analysis, of which 686 (43.86%) respondents were Turkish-Dutch women and 878 respondents (56.14%) were Moroccan-Dutch women: 793 (50.7%) respondents in the control group (350/793, 44.1% Turkish and 443/793, 55.9% Moroccan) and 771 respondents (49.3%) in the intervention group (336/771, 43.6% Turkish and 435/771, 56.4% Moroccan; Figure 2).

### Sample Characteristics

The final sample (n=1564) consisted of 686 (43.86%) Turkish-Dutch women and 878 (56.14%) Moroccan-Dutch women (Table 1). Most respondents in both groups were aged between 30 and 39 years and were highly educated (295/686, 43% and 454/878, 51.7%, respectively), and 8% (55/686) and 12% (105/878) of the respondents had no official or primary education, respectively. Overall, 59.9% (411/686) of the Turkish women and 56.4% (495/878) of the Moroccan women were second-generation immigrants. Their social network on the web (ie, other Turkish- or Moroccan-Dutch women aged 30-60 years) was mostly between 11 and 49 women, and 3% of the respondents had no social network on the web.

**Table 1:** Sample characteristics of the Turkish- and Moroccan-Dutch respondents^a^.

Characteristics			Turkish (n=686), n (%)		National data proportions^a^ (Turkish), %		Moroccan (n=878), n (%)		National data proportions^a^ (Moroccan), %
**Age (years)**									
	30-39	418 (60.9)		36		455 (51.8)		40	
	40-49	189 (27.6)		37		328 (37.4)		37	
	50-60	92 (13.4)		27		95 (10.8)		23	
	Missing value	0 (0)		N/A^b^		0 (0)		N/A	
**Educational level**									
	No official education or primary school	82 (12.0)		44^c^		68 (7.7)		43^c^	
	Secondary school	110 (16.0)		N/A		136 (15.5)		N/A	
	Vocational education	198 (28.9)		33		219 (24.9)		39	
	Higher education	295 (43.0)		23		454 (51.7)		18	
	Missing value	1 (0)		N/A		1 (0)		N/A	
**Generation**									
	First	275 (40.1)		72		383 (43.6)		73	
	Second	411 (59.9)		28		495 (56.4)		27	
	Missing value	0 (0)		N/A		0 (0)		N/A	
**Size of their social network on the web**									
	0	17 (2.5)		N/A		24 (2.7)		N/A	
	1-10	180 (26.2)		N/A		160 (18.2)		N/A	
	11-49	273 (39.8)		N/A		412 (46.9)		N/A	
	50-99	96 (14.0)		N/A		157 (17.9)		N/A	
	100-249	74 (10.8)		N/A		91 (10.4)		N/A	
	250-499	33 (4.8)		N/A		29 (3.3)		N/A	
	≥500	13 (1.9)		N/A		5 (1.0)		N/A	
	Missing value	0 (0)		N/A		0 (0)		N/A	
**Language in which the questionnaire was completed**									
	Dutch	432 (63.0)		N/A		758 (86.3)		N/A	
	Turkish or Arabic	234 (34.1)		N/A		2 (0)		N/A	
	Missing value (due to technical failure)	20 (2.9)		N/A		118 (13.4)		N/A	
**Previous CC^d^ screening participation**									
	Every 5 years	305 (44.5)		N/A		433 (49.3)		N/A	
	Not every 5 years	106 (15.5)		N/A		125 (14.2)		N/A	
	Never	275 (40.1)		N/A		320 (36.4)		N/A	
	Missing value	0 (0)		N/A		0 (0)		N/A	

^a^Extracted from databases [18-20].

^b^N/A: not applicable.

^c^Includes no official education, primary school, and secondary school.

^d^CC: cervical cancer.

In total, 40.1% (275/686, Turkish) and 36.4% (320/878, Moroccan) of the respondents indicated that they had never participated in CC screening before, 44.5% (305/686, Turkish) and 49.3% (433/878, Moroccan) of the respondents reported to have participated in CC screening once every 5 years, and 15.5% (106/686, Turkish) and 14.2% (125/878, Moroccan) of the respondents participated irregularly. The respondents represented a wide geographic area across the Netherlands (Figures S1 and S2 in Multimedia Appendix 1).

Among the Turkish-Dutch women, 33.1% (116/350) of the control respondents and 40.5% (136/336) of the intervention respondents viewed the brochure (not statistically significant). Of the intervention respondents, 96.1% (323/336) of the respondents viewed the CSEV. Among the Moroccan-Dutch women, 28.2% (125/443) of the control respondents and 37.9% (165/435) of the intervention respondents viewed the brochure (*P*=.003). Of the intervention respondents, 84.4% (367/435) of the respondents viewed the CSEV.

### Knowledge of CC Screening

Turkish-Dutch respondents with sufficient knowledge of CC screening increased from 54.6% (191/350) to 68.3% (239/350) in the control group (+13.7% absolute change; *P*<.001) and from 49.1% (165/336) to 63.7% (214/336) in the intervention group (+14.6%; *P*<.001). Moroccan-Dutch respondents with sufficient knowledge increased from 61.4% (272/443) to 78.8% (349/443) in the control group (+17.4%; *P*<.001) and from 65.7% (286/435) to 77.5% (337/435) in the intervention group (+11.8%; *P*<.001). In terms of knowledge, the CSEV did not show a significant effect above the information brochure for either group (see Tables S1 and S2 in Multimedia Appendix 1).

### Attitude Toward CC Screening

Turkish-Dutch respondents with a positive attitude toward CC screening decreased from 70% (245/350) to 67.1% (235/350) in the control group (−2.9%; not statistically significant) and from 66.7% (224/336) to 66.4% (223/336) in the intervention group (−0.3%; not statistically significant). Moroccan-Dutch respondents with a positive attitude increased from 64.6% (286/443) to 68.4% (303/443) in the control group (+3.8%; not statistically significant) and from 65.1% (283/435) to 74.3% (323/435) in the intervention group (+9.2%; *P*=.004). Overall, there was no added effect of the CSEV on the attitude toward CC screening among Turkish-Dutch women (*P*=.89; Table S3 in Multimedia Appendix 1). We found that Moroccan-Dutch women in the intervention group more often had a positive attitude toward CC screening compared with the control group, although this difference was not statistically significant (*P*=.07; Table S4 in Multimedia Appendix 1). Moroccan-Dutch women in the intervention group who had never participated in CC screening before had significantly more often a positive attitude toward CC screening compared with the control group (*P*=.01).

### Intention and IDM Regarding CC Screening Participation

Both the control and intervention groups had more often a positive intention after consulting the brochure or the brochure and CSEV in both Turkish- and Moroccan-Dutch women (Table 2). An increase was observed among Turkish-Dutch women from 78.3% (274/350) to 82.6% (289/350) in control respondents (+4.3%; not statistically significant) and from 79.2% (266/336) to 84.5% (284/336) in intervention respondents (+5.3%; not statistically significant). The same holds true for Moroccan-Dutch women: from 79.9% (354/443) to 86% (381/443) in control respondents (+6.1%; *P*=.02) and from 80% (348/435) to 86.9% (378/435) in intervention respondents (+6.9%; *P*=.008). However, the CSEV did not have a statistically significant added effect above the brochure in terms of intention.

In general, women made more often an informed decision after the control or intervention condition among Turkish- and Moroccan-Dutch women (Table 2). Of the control respondents, IDM increased from 38.6% (135/350) to 44.3% (155/350) in Turkish-Dutch women (+5.7%; not statistically significant) and from 43.8% (194/443) to 53.7% (238/443) in Moroccan-Dutch women (+9.9%; *P*=.004). The same holds true for intervention respondents; we saw an increase in IDM from 34.5% (116/336) to 42.9% (144/336) in Turkish-Dutch women (+8.4%; *P*=.03) and from 44.6% (194/435) to 58.9% (256/435) in Moroccan-Dutch women (+14.3%; *P*<.001). However, the CSEV did not have a statistically significant added effect above the brochure in terms of IDM (Tables 2 and 3).

**Table 2:** Intention and informed decision-making (IDM) regarding cervical cancer (CC) screening participation in the control and intervention groups, before and after reading the brochure (control) or reading the brochure and watching the culturally sensitive educational video (intervention).

Characteristics			Population											
			Turkish-Dutch women							Moroccan-Dutch women				
			Control group (n=350), n (%)		Intervention group (n=336), n (%)		*P* value		Control group (n=443), n (%)			Intervention group (n=435), n (%)		*P* value
**Intention to participate in CC screening (before)**														
	Positive	274 (78.3)		266 (79.2)		.85		354 (79.9)			348 (80.0)		>.99	
	Neutral	60 (17.1)		58 (17.3)		>.99		65 (14.7)			68 (15.6)		.76	
	Negative	16 (4.6)		12 (3.6)		.64		24 (5.4)			19 (4.4)		.57	
**Intention to participate in CC screening (after)**														
	Positive	289 (82.6)		284 (84.5)		.56		381 (86.0)			378 (86.9)		.77	
	Neutral	48 (13.7)		41 (12.2)		.63		45 (10.2)			41 (9.4)		.80	
	Negative	13 (3.7)		11 (3.3)		.92		17 (3.8)			16 (3.7)		>.99	
**IDM (before)**														
	Yes	135 (38.6)		116 (34.5)		.31		194 (43.8)			194 (44.6)		.86	
	No	215 (61.4)		220 (65.5)		N/A^a^		249 (56.2)			241 (55.4)		N/A	
**IDM (after)**														
	Yes	155 (44.3)		144 (42.9)		.76		238 (53.7)			256 (58.9)		.14	
	No	195 (55.7)		192 (57.1)		N/A		205 (46.3)			179 (41.1)		N/A	

^a^N/A: not applicable.

**Table 3:** Informed decision-making regarding cervical cancer (CC) screening participation in the control and intervention groups, after reading the brochure (control) or reading the brochure and watching the culturally sensitive educational video (intervention).

Characteristics			Population											
			Turkish-Dutch women							Moroccan-Dutch women				
			Control group (N=350), n (%); uninformed^a^		Intervention group (N=336), n (%); uninformed^a^		*P* value		Control group (N=443), n (%); uninformed^a^			Intervention group (N=435), n (%); uninformed^a^		*P* value
**Age (years)**														
	30-39	124 (35.4)		112 (33.3)		.62		107 (24.2)			89 (20.5)		.22	
	40-49	47 (13.4)		52 (15.5)		.51		72 (16.3)			62 (14.3)		.47	
	50-60	24 (6.9)		28 (8.3)		.56		26 (5.9)			28 (6.4)		.83	
**Educational level**														
	No official education or primary school	23 (6.6)		32 (9.5)		.20		18 (4.1)			25 (5.7)		.32	
	Secondary school	29 (8.3)		39 (11.6)		.18		34 (7.7)			25 (5.7)		.31	
	Vocational education	62 (17.7)		54 (16.1)		.64		54 (12.2)			44 (10.1)		.39	
	Higher education	80 (22.9)		67 (19.9)		.40		99 (22.3)			85 (19.5)		.35	
**Generation**														
	First	74 (21.1)		89 (26.5)		.12		96 (21.7)			87 (20.0)		.60	
	Second	121 (34.6)		103 (30.7)		.31		109 (24.6)			92 (21.1)		.26	
**Previous** **CC** **screening participation**														
	Every 5 years	58 (16.6)		71 (21.1)		.15		70 (15.8)			56 (12.9)		.25	
	Not every 5 years	37 (10.6)		31 (9.2)		.64		26 (5.9)			31 (7.1)		.54	
	Never	100 (28.6)		90 (26.8)		.66		109 (24.6)			92 (21.1)		.26	

^a^Uninformed: The number of women classified as being uninformed.

### Self-sampling

No statistically significant differences were found in awareness, perceptions, and intention regarding self-sampling when comparing the control and intervention groups among Turkish-Dutch women (Table S9 in Multimedia Appendix 1).

More Moroccan-Dutch respondents thought that self-sampling was easy to perform in the intervention group than in the control group (284/435, 65.3% vs 252/443, 56.9%; *P*=.04). In addition, fewer respondents in the intervention group thought that self-sampling would be painful compared with the control group (59/435, 13.6% vs 82/443, 18.5%; *P*=.05; Table S10 in Multimedia Appendix 1).

## Discussion

### Principal Findings

This study evaluated the effect of a CSEV on knowledge, attitude, intention, and IDM regarding CC screening among Turkish- and Moroccan-Dutch women aged 30 to 60 years. The CSEV was watched far more often than the brochure was read when both were offered together, and the intervention group who watched the video also studied the brochure more often than the control group did. The brochure had a significant positive influence on IDM, whereas the CSEV had an added effect on the attitude toward CC screening, especially in Moroccan-Dutch women. These women more often had a positive attitude toward CC screening compared with the control group who had read only the brochure. This was especially the case among women who had never participated in CC screening before. On the basis of the open-field comments of Turkish-Dutch respondents, we think we can explain why this effect was not visible in this group. It appeared that some of the Turkish-Dutch respondents were offended by the fact that in the Turkish video, the actress who played having a negative screening attitude was wearing a headscarf.

### Comparison With Prior Work

In line with our results in the control group, a previous study among Dutch women invited for breast cancer screening also found that reading the brochure enhanced IDM [21]. Earlier randomized controlled trials that strived to enhance IDM regarding cancer screening often developed a decision aid, in which information was presented differently compared with the standard letter or brochure [22-26]. These studies tended to target knowledge instead of the attitudes we aimed at. In line with our study, an earlier randomized controlled trial in Germany among all targeted women without a Turkish migration background also compared the standard information brochure for breast cancer screening with a newly developed decision aid [27]. In contrast to our study, more respondents in the intervention group were knowledgeable compared with those in the control group. This seems to be related to the fact that the same information was presented in both the groups, but only visually, instead of textually, in the intervention group versus the control group. We did not include any factual medical information in the CSEV and did not target women’s knowledge. In the United Kingdom, a similar intervention study regarding participation in lung cancer screening among smokers also used a video and found that it improved knowledge and reduced decisional conflict [28]. However, this video was also targeted at increasing knowledge instead of improving screening attitudes.

### Implications for Practice and Policy

We recommend developing videos that incorporate information provided in the current brochure, as many Turkish- and Moroccan-Dutch women do not read the brochure (thoroughly) or are simply unable to read it [5]. In line with this study, a video has been shown to be more engaging and attractive than textual information [29]. Considering that approximately one-third of the control group consulted the brochure, the effect of the brochure on IDM might be greater if the brochure was studied more often and in more detail. We expect that in the context of this study, respondents were more likely to read the brochure (intensively) than those who received it with the invitation (ie, the Hawthorne effect). Therefore, we recommend presenting the CSEV to all women through the invitation letter, for example, using a weblink or a QR code, so that the CSEV and all other web-based materials can be accessed easily. We propose to consider using the CSEV in mosques, community centers, and educational meetings regarding (women’s) health for women with limited digital skills. Other options include distributing the CSEV in women’s groups on Facebook or broadcasting the CSEV on a loop in the waiting room at the GP’s office.

Women are invited to undergo CC screening every 5 years and might not be interested to search for or gather information every time they are invited. Therefore, in addition to evaluating different modes of delivering visual information, we recommend that research be performed on the use of different distribution channels to reach uninformed women, such as social media and involvement of influencers, key figures, informants, and close-knit community groups that were used in this study.

In October 2021, the Dutch Health Council recommended offering self-sampling as an equivalent alternative to clinician-based sampling and sending the self-sampling kit together with the invitation [30]. Owing to the CSEV, more Moroccan-Dutch respondents thought that self-sampling was easy to perform and fewer respondents thought that self-sampling would be painful. Therefore, sending the self-sampling kit with the invitation should concur with implementing our CSEV. Overall, as a short intervention that is easily implemented, our CSEV represents an efficient way to enhance screening attitudes and facilitate IDM among immigrant women.

### Strengths and Limitations

One major strength of this study was its design as a randomized intervention study. Worldwide, this study also had one of the largest samples successfully recruited using web-based RDS [31]. In addition, our CSEVs were systematically developed based on extensive qualitative and quantitative research among Turkish- and Moroccan-Dutch women [5]. The brochure that we used in our study was sent to all women aged 30 to 60 years by the regional screening organizations. This brochure has been used in practice since November 2016 and is considered “usual care,” and it openly discusses potential benefits and harms of CC screening. Therefore, we deliberately used the CSEV as an addition to the brochure to facilitate one’s individual thinking process and/or discussion with other women and not as a replacement intervention. Our CSEV can now be easily added to the existing invitation materials. More importantly, our CSEV includes other more affective aspects, which are not incorporated in the brochure but are needed for the Turkish- and Moroccan-Dutch women to be able to make a conscious decision on their CC screening participation [5].

However, a number of limitations should also be addressed. First, owing to the web-based delivery of the questionnaire, we sampled a greater number of women who were aged 30 to 39 years, were second-generation immigrants, and were highly educated Turkish- and Moroccan-Dutch women compared with the national data set of 2020 of Statistics Netherlands [18-20]. However, the 2 randomized groups were comparable, and 12% (82/686) and 8% (70/878) of the Turkish- and Moroccan-Dutch respondents reported no official education or completed primary school, respectively. In addition, regarding previous CC screening participation, we did find similar rates of at least one participation in CC screening of 60% (412/686) and 64% (562/878) of the respondents in Turkish- and Moroccan-Dutch women versus 64% and 53% of the respondents, respectively, in previous reports [6].

Second, the time elapsed between the previous screening invitation and the questionnaire administration, which varied largely among our respondents, might have affected the experienced relevance of the decision-making questions and the previously existing knowledge. However, this heterogeneity is likely to play a similar role (if it does at all) in both the control and intervention groups because of the randomization performed.

Third, the women who participated in our study might have been different from those who did not participate in the study. For example, they could be more interested in CC screening as a topic and be more informed about it than nonparticipating women. Nevertheless, as we used incentives for successful peer recruitment, this might also have been the reason that some respondents participated in the study rather than being interested in CC screening. In addition, this possible selection bias is likely to be present in both the control and intervention groups and should not affect the evaluation of the CSEV.

Fourth, our knowledge construct contained only some facts about CC screening (ie, the process after a negative or positive test result and the possibility of false-positive test results). Although these have been carefully selected, they do not cover the entire spectrum of decision-relevant information (eg, hrHPV as the causative agent of CC and its transmission route) and can only indicate some deficits. Because of the use of RDS, and thus requesting women to successfully recruit others, we aimed to burden the respondents as less as possible and, therefore, kept the questionnaire as short as possible.

Fifth, health literacy (ie, the degree to which individuals have the ability to find, understand, and use information and services to take informed health-related decisions and actions for themselves and others) is crucial to make informed health-related decisions. The immigrants are less capable of applying IDM, as they have lower health literacy levels compared with nonimmigrants [32]. It would have been interesting to assess health literacy levels of individuals to compare the effect of our CSEV among those with limited and adequate health literacy levels.

Sixth, to further explore the differences found between Turkish- and Moroccan-Dutch women and their attitudes, thoughts, and views regarding the current information brochure and the CSEV, it would have been highly relevant to conduct follow-up interviews or focus groups, shortly after the end of our randomized intervention study.

Finally, we based the content of the CSEVs on our earlier conducted focus groups among offline-recruited Turkish- and Moroccan-Dutch women [5]. Because of the measures taken for the COVID-19 pandemic (eg, nationwide lockdowns), we were unable to approach potential respondents face-to-face and recruit them offline. The respondents were also unable to recruit peers offline unless they were household members. This resulted in a web-based–only, relatively young, mostly second-generation sample of Turkish- and Moroccan-Dutch women. It would be highly relevant to evaluate the CSEVs in an offline setting, which is comparable with our previous study [5]. We believe that CSEVs could affect IDM (greater) in such a setting for which the CSEVs were tailored during the development process.

### Conclusions

This randomized intervention study has demonstrated that a CSEV positively affected CC screening attitudes, especially among Moroccan-Dutch women. Women who were offered both the brochure and CSEV consulted the brochure more often than those who received the brochure only. The CSEV was also watched far more often than the brochure was read. Therefore, the CSEV can be widely distributed through offline and web-based channels, in addition to the current information materials.

## Appendix

Multimedia Appendix 1 Supplementary tables and figures.

Multimedia Appendix 2 CONSORT-eHEALTH Checklist (V 1.6.1).

## Abbreviations

CC: cervical cancer
CONSORT: Consolidated Standards of Reporting Trials
CSEV: culturally sensitive educational video
GP: general practitioner
hrHPV: high-risk human papillomavirus
IDM: informed decision-making
RDS: respondent-driven sampling
