# Supplementary material for: Evaluation of a Web-Based Culturally Sensitive Educational Video to Facilitate Informed Cervical Cancer Screening Decisions Among Turkish- and Moroccan-Dutch Women Aged 30 to 60 Years: Randomized Intervention Study
Source: J Med Internet Res. 2022 Oct 26;24(10):e35962. doi: 10.2196/35962 (PMC9647450; doi:10.2196/35962)
Supplement: Multimedia Appendix 1 [file jmir_v24i10e35962_app1.docx]

**SUPPLEMENTARY TABLES AND FIGURES**

*Evaluation of a culturally sensitive educational video to facilitate informed cervical cancer screening decisions among Turkish- and Moroccan-Dutch women:*

*A randomized intervention study*

**Table of contents**

| **Content** | **Page(s)** |
| --- | --- |
| Supplement 1. Questionnaire (in Dutch) | 3-15 |
| Figure S1. Geographical distribution of Turkish-Dutch respondents | 16 |
| Figure S2. Geographical distribution of Moroccan-Dutch respondents | 17 |
| Table S1. Knowledge of CC screening among the control and intervention Turkish-Dutch respondents, after the brochure (control) or brochure and CSEV (intervention) | 18 |
| Table S2. Knowledge of CC screening among the control and intervention Moroccan-Dutch respondents, after the brochure (control) or brochure and CSEV (intervention) | 18 |
| Table S3. Attitude towards CC screening participation among the control and intervention Turkish-Dutch respondents, after the brochure (control) or brochure and CSEV (intervention) | 19 |
| Table S4. Attitude towards CC screening participation among the control and intervention Moroccan-Dutch respondents, after the brochure (control) or brochure and CSEV (intervention) | 19 |
| Table S5. Pre- and post-IDM in the control group, among Turkish-Dutch women | 20 |
| Table S6. Pre- and post-IDM in the intervention group, among Turkish-Dutch women | 20 |
| Table S7. Pre- and post-IDM in the control group, among Moroccan-Dutch women | 20 |
| Table S8. Pre- and post-IDM in the intervention group, among Moroccan-Dutch women | 20 |
| Table S9. Awareness, perceptions, and intention regarding self-sampling, after the brochure (control) or brochure and CSEV (intervention), among Turkish-Dutch women | 21 |
| Table S10. Awareness, perceptions, and intention regarding self-sampling, after the brochure (control) or brochure and CSEV (intervention), among Moroccan-Dutch women | 21 |

**Supplement 1. Questionnaire (in Dutch)**


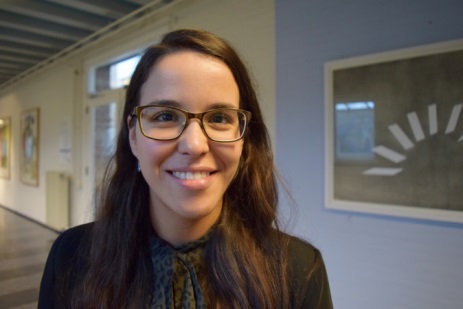
**Salaam alaikoum beste mevrouw**,

Mijn naam is Nora Hamdiui. Ik werk bij het Radboud ziekenhuis.

**Waarom krijgt u deze vragenlijst?**

**1**

Alle vrouwen van 30 tot 60 jaar krijgen een uitnodiging om mee te doen aan het bevolkingsonderzoek baarmoederhalskanker. Dit onderzoek gaat over kanker aan de onderkant van de baarmoeder en wordt vaak bij de huisarts gedaan.

[
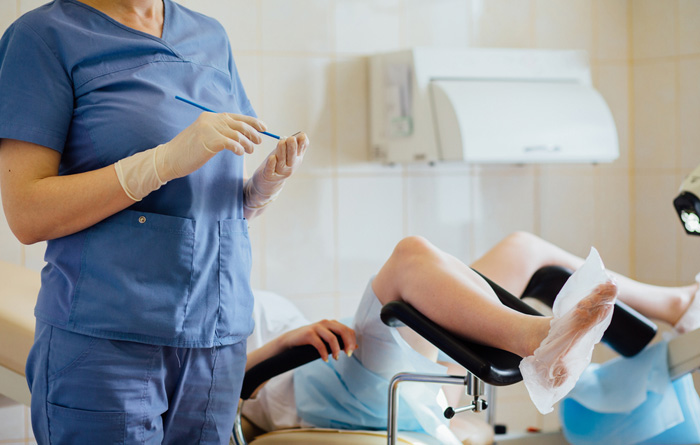
](https://www.google.com/url?sa=i&url=https%3A%2F%2Fwww.mamsatwork.nl%2Fuitstrijkje-laten-maken%2F&psig=AOvVaw0lBobKLt9gGbKlCBirdfgI&ust=1584109960130000&source=images&cd=vfe&ved=0CAIQjRxqFwoTCNif182TlegCFQAAAAAdAAAAABAT)[
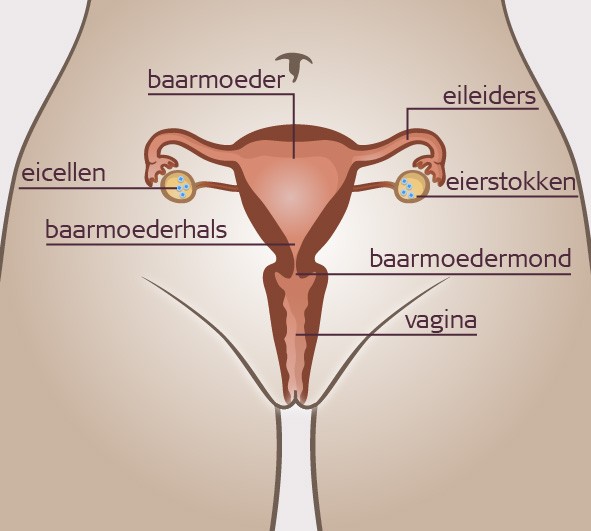
](https://www.google.com/url?sa=i&url=https%3A%2F%2Fwww.allesoverseks.be%2Fbaarmoeder&psig=AOvVaw2eA4ZVoFME2S-SVu8Xg1aY&ust=1584109508653000&source=images&cd=vfe&ved=0CAIQjRxqFwoTCKi8vfaRlegCFQAAAAAdAAAAABAE)

Sommige vrouwen doen niet mee aan het bevolkingsonderzoek baarmoederhalskanker. Ik wil graag weten waarom vrouwen niet meedoen, maar ook waarom vrouwen wel meedoen.

Daarom stel ik u graag vragen over wat u van dit onderzoek vindt. U kunt deze vragen invullen als u wel het onderzoek heeft gedaan. En ook als u nog geen onderzoek heeft laten doen.

Met uw antwoorden maken we betere informatie. De betere informatie kan vrouwen helpen om te kiezen of ze mee gaan doen aan het bevolkingsonderzoek.

[
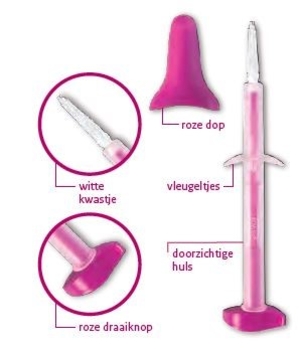
](https://www.google.com/url?sa=i&url=https%3A%2F%2Folijf.nl%2Fover-olijf%2Fnieuws%2Fnieuws-page%2Farticle%2Fpreventieweek-baarmoederhalskanker-zelfafnameset%2F&psig=AOvVaw1ipGHhzICjEa0QPe6fJgad&ust=1582731063542000&source=images&cd=vfe&ved=0CAIQjRxqFwoTCJi71OiC7ecCFQAAAAAdAAAAABAE)**Daarom heb ik uw hulp nodig!**

**2**

**Wilt u de vragen op de volgende pagina’s invullen?**

Het invullen duurt ongeveer 15 minuten. De vragen gaan over het onderzoek bij de huisarts. Je kan dit onderzoek ook thuis doen zonder hulp van de huisarts. Dat heet de zelfafnameset. Daarover stel ik ook vragen.

**Cadeaubon voor het doorsturen**

**3**

[
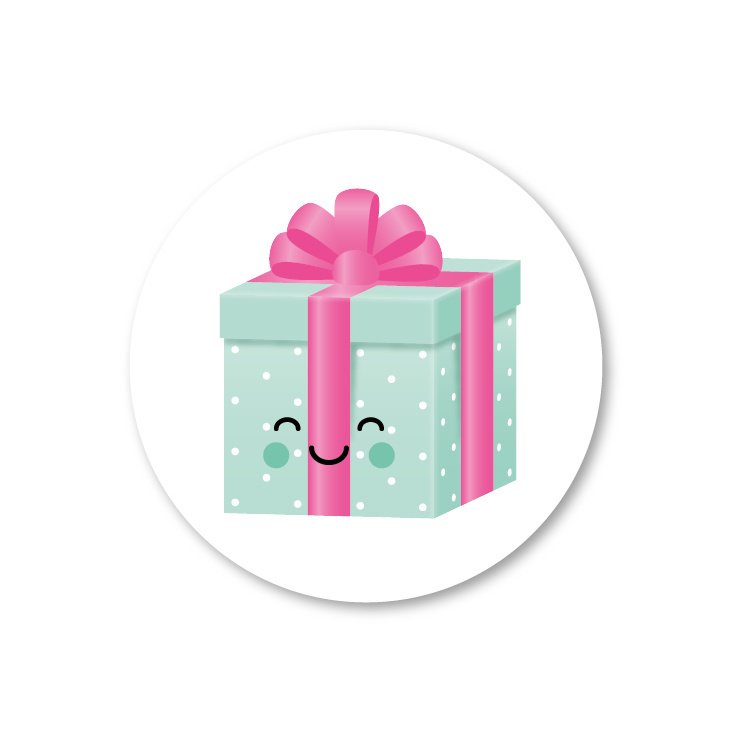
](http://www.google.com/url?sa=i&rct=j&q=&esrc=s&source=images&cd=&cad=rja&uact=8&ved=2ahUKEwjV-oPmib_lAhXLJVAKHTr2D-EQjRx6BAgBEAQ&url=/url?sa%3Di%26rct%3Dj%26q%3D%26esrc%3Ds%26source%3Dimages%26cd%3D%26ved%3D2ahUKEwjV-oPmib_lAhXLJVAKHTr2D-EQjRx6BAgBEAQ%26url%3Dhttps://www.kadootjes-online.nl/a-51275679/kado-stickers/5-stickers-kadootje-groen-roze/%26psig%3DAOvVaw3v0xM1CAy7K8wUHuB_Qtyp%26ust%3D1572356265259346&psig=AOvVaw3v0xM1CAy7K8wUHuB_Qtyp&ust=1572356265259346)Het is belangrijk dat veel vrouwen deze vragen invullen. Dan maken we de informatie over het onderzoek beter. Kent u andere Marokkaans-Nederlandse vrouwen tussen de 30 en 60 jaar? Stuur deze vragenlijst dan aan hen door en dan krijgt u een cadeaubon.

**Wat moet u doen om een cadeaubon te krijgen?**

1. Vul deze vragenlijst in.
2. Vraag 2 vrouwen deze vragenlijst in te vullen.

Als zij de vragenlijst invullen, krijgt u een **cadeaubon van € 10.** U krijgt alleen een cadeaubon als 2 vrouwen de vragenlijst invullen. 1 vrouw is niet genoeg.

**Wilt u de vragen invullen?**

- **Ja**

Vul hieronder uw e-mailadres in:

[………………………………………] (e-mailadres verplicht invullen)

- **Nee**

**Uw antwoorden**

Alles wat u invult in deze vragenlijst blijft geheim. Alleen ik en mijn collega’s zien uw antwoorden. Wilt u meer lezen over hoe we uw antwoorden geheim houden? Klik dan op deze link: [www.rivm.nl/feminine](http://www.rivm.nl/feminine).

Hartelijk bedankt / Shokran voor het invullen!

**BarakAllahu feek**

Nora Hamdiui

**Niet verplicht**

U hoeft de vragenlijst niet in te vullen. U mag zelf kiezen. U mag altijd stoppen met invullen.

**Vragen**

Wilt u nog iets vragen over deze vragenlijst? Of heeft u hulp nodig bij het invullen? Ik wil u graag helpen.

U kunt mij bellen op dit nummer: **030 274 2884**. Ik spreek Nederlands en Berbers en kan u in het Nederlands en Berbers helpen met de vragen.

Of stuur mij een e-mail naar: [feminine@rivm.nl](mailto:feminine@rivm.nl).

**Op de volgende pagina beginnen de vragen.**

**Beantwoord de vragen door uw antwoorden aan te klikken.**

**Vul alle vragen in.**

**Ik stel eerst een aantal vragen over uzelf…**

1. Wat is uw geboortemaand en -jaar?

**- 1 9**

2. Wat zijn de vier cijfers van uw postcode?

3. Welke opleiding heeft u afgemaakt?

- Geen opleiding
- Basisschool
- Middelbare school

*Attestation du Baccalauréat*

- Technisch of beroepsonderwijs (LTS, MTS, MBO)

*Diplôme de Technicien*

- Hoger beroepsonderwijs (HBO), universiteit (WO)

*Diplôme de Technicien Spécialisé, Brevet de Technicien Supérieur, Diplôme Universitaire de Technologie, Diplôme d’Ingénieur d’ Application, License, Master*

- Anders, namelijk: ……………………………………………………………….

4. In welk land bent u geboren?

- Marokko
- Nederland
- Anders, vul hier in waar: …….…….…….…….

5. In welk land is uw moeder geboren?

- Marokko
- Nederland
- Anders, vul hier in waar: …….…….…….…….

6. In welk land is uw vader geboren?

- Marokko
- Nederland
- Anders, vul hier in waar: …….…….…….…….

[
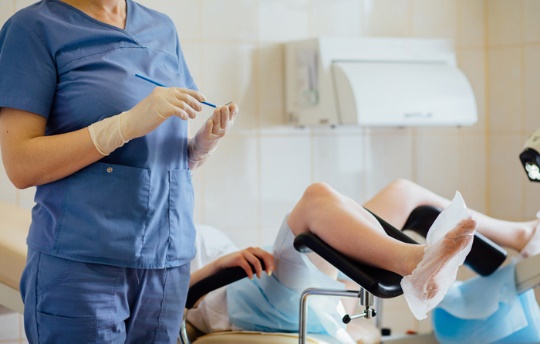
](https://www.google.com/url?sa=i&url=https%3A%2F%2Fwww.mamsatwork.nl%2Fuitstrijkje-laten-maken%2F&psig=AOvVaw0lBobKLt9gGbKlCBirdfgI&ust=1584109960130000&source=images&cd=vfe&ved=0CAIQjRxqFwoTCNif182TlegCFQAAAAAdAAAAABAT)De volgende vragen gaan over het **bevolkingsonderzoek baarmoederhalskanker**. Bij dit onderzoek maakt de huisarts of doktersassistente een uitstrijkje. Met een borsteltje haalt hij of zij wat baarmoederslijm van de baarmoederhals af. Of u doet het onderzoek zelf thuis, zonder hulp van iemand anders.

7. Heeft u **wel eens** meegedaan aan het bevolkingsonderzoek baarmoederhalskanker?

- Ja, ik doe elke 5 jaar het onderzoek
- Ja, maar ik doe niet elke 5 jaar het onderzoek
- Nee, ik heb nog nooit meegedaan 🡪 **Ga naar vraag 9**

8. Deed u **ooit** mee aan het bevolkingsonderzoek baarmoederhalskanker door het onderzoek zelf thuis te doen?

- Ja
- Nee

De vragen hieronder gaan over **wat u weet over het bevolkingsonderzoek** **baarmoederhalskanker**. Als u het antwoord niet weet, klik dan op: **Ik weet het niet**.

9. Wat moet u doen als de uitslag van het onderzoek **goed** is?

- Niks, ik krijg over 5 jaar vanzelf een brief thuis voor het uitstrijkje
- Ik moet een extra onderzoek doen
- Ik moet mij zelf aanmelden voor het volgende uitstrijkje over 5 jaar
- Ik weet het niet

10. Wat moet u doen als de uitslag van het onderzoek **niet goed** is?

**Bij deze vraag mag u meer antwoorden geven.**

- Er gebeurt niks
- Ik moet nog een uitstrijkje laten doen
- Ik moet naar het ziekenhuis voor meer onderzoek
- Ik krijg medicijnen
- Ik weet het niet

11. Als de uitslag van het onderzoek **niet goed** is, heeft die persoon dan baarmoederhalskanker?

- Ja
- Nee
- Ik weet het niet

De volgende vragen gaan over **wat u vindt van het bevolkingsonderzoek baarmoederhalskanker**.

Klik uw antwoord aan.

12. Vindt u het **nuttig** om het onderzoek te laten doen?

- Ja
- Ik weet het niet
- Nee

13. Bent u **bang** om het onderzoek te laten doen?

- Ja
- Ik weet het niet
- Nee

14. Vindt u het **spannend** om het onderzoek te laten doen?

- Ja
- Ik weet het niet
- Nee

15. **Schaamt** u zich om het onderzoek te laten doen?

- Ja
- Ik weet het niet
- Nee

16. Vindt u dat u genoeg **privacy** heeft tijdens het onderzoek?

*Met privacy bedoel ik dat u alleen met de huisarts of doktersassistente bent en dat er gordijnen zijn waar u uw kleren kunt uit doen.*

- Ja
- Ik weet het niet
- Nee

17. Vindt u dat het onderzoek **pijn** doet?

- Ja
- Ik weet het niet
- Nee

18. Denkt u dat u meer weet over hoe **gezond** u bent als u het onderzoek laat doen?

- Ja
- Ik weet het niet
- Nee

19. Denkt u dat u geen (zware) **behandeling of operatie** hoeft als u het onderzoek laat doen?

- Ja
- Ik weet het niet
- Nee

20. Denkt u dat u een kleinere kans hebt om **dood te gaan** door baarmoederhalskanker als u het onderzoek laat doen?

- Ja
- Ik weet het niet
- Nee

21. Laat u het onderzoek doen, omdat het **volgens uw geloof belangrijk** is om goed voor uw lichaam te zorgen?

- Ja
- Ik weet het niet
- Nee

22. Gaat u **de volgende keer** als u de brief krijgt, het onderzoek laten doen?

- Ja
- Ik weet het niet
- [
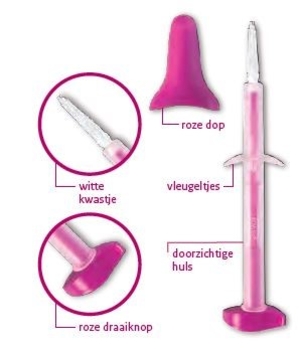
](https://www.google.com/url?sa=i&url=https%3A%2F%2Folijf.nl%2Fover-olijf%2Fnieuws%2Fnieuws-page%2Farticle%2Fpreventieweek-baarmoederhalskanker-zelfafnameset%2F&psig=AOvVaw1ipGHhzICjEa0QPe6fJgad&ust=1582731063542000&source=images&cd=vfe&ved=0CAIQjRxqFwoTCJi71OiC7ecCFQAAAAAdAAAAABAE)Nee

U kunt ook thuis een onderzoek doen. Dat kunt u zelf doen met de zelfafnameset. Dat kan sinds 2017.

23. Weet u wat een zelfafnameset is?

- Ja
- Nee

De volgende vragen gaan over **wat u denkt van de zelfafnameset**.

Klik uw antwoord aan.

24. Denkt u dat het doen van de zelfafnameset **makkelijk** is?

- Ja
- Ik weet het niet
- Nee

25. Denkt u dat de zelfafnameset **pijn** doet?

- Ja
- Ik weet het niet
- Nee

26. Denkt u dat u de zelfafnameset zelf **goed kunt doen**?

- Ja
- Ik weet het niet
- Nee

27. Gelooft u de **uitslag** van de zelfafnameset?

- Ja
- Ik weet het niet
- Nee

28. Wilt u **de volgende keer** een **zelfafnameset** doen voor het onderzoek over baarmoederhalskanker?

- Ja
- Ik weet het niet
- Nee

Op de volgende pagina kunt u een informatiefolder zien. Deze folder krijgt u ook bij de uitnodiging voor het bevolkingsonderzoek baarmoederhalskanker.

Lees deze rustig door.

Ik laat ook een filmpje zien over [naam hoofdpersoon]. Zij moet kiezen of ze wel of niet mee gaat doen aan het bevolkingsonderzoek.

Bekijk dit filmpje.

Daarna krijgt u bijna dezelfde vragen die u tot nu toe ingevuld heeft. Als u de hele vragenlijst invult, leren wij hoe we de informatie in de toekomst beter kunnen maken.

Ik wil graag weten waarom Nederlanders met een migrantenachtergrond minder vaak meedoen met het onderzoek. Bijvoorbeeld Marokkaans-Nederlandse vrouwen. Daarom wil ik weten hoeveel Marokkaans-Nederlandse vrouwen* deze vragenlijst gaan invullen. Daarom stel ik de volgende vraag.

*Met Marokkaans-Nederlandse vrouwen bedoel ik vrouwen die in Marokko geboren zijn of een moeder en/of vader hebben die in Marokko is geboren.

49. Hoeveel **Marokkaans-Nederlandse vrouwen tussen de 30 en 60 jaar** kent u die deze online vragenlijst kunnen invullen?

|  | **Aantal** |
| --- | --- |
| Familie |  |
| Vriendinnen |  |
| Collega’s of vrouwen van mijn studie |  |

Dit is het einde van de vragenlijst. Hartelijk bedankt / Shokran voor het invullen! BarakAllahu feek.

50. Wilt u meer vragenlijsten voor Marokkaans-Nederlandse vrouwen invullen?

- Ja, mail mij meer informatie
- Nee


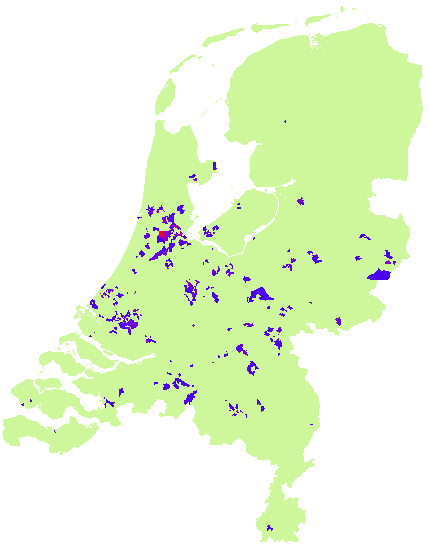


**Figure S1. Geographical distribution of Turkish-Dutch respondents**

A map showing the geographical area of respondents was created with a shapefile that was extracted from GADM, an online geographic database of global administrative areas (1).


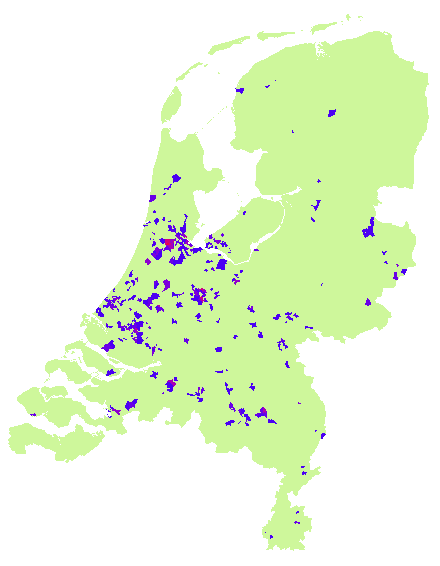


**Figure S2. Geographical distribution of Moroccan-Dutch respondents**

A map showing the geographical area of respondents was created with a shapefile that was extracted from GADM, an online geographic database of global administrative areas (1).

**Table S1. Knowledge of CC screening among the control and intervention Turkish-Dutch respondents, after the brochure (control) or brochure and CSEV (intervention)**

| **Characteristic** | | **Control group**  **N = 350** | **Intervention group**  **N = 336** | **P value** |
| --- | --- | --- | --- | --- |
|  |  | **Sufficient knowledge** | **Sufficient knowledge** |  |
| **Total group** | | 239 (68.3%) | 214 (63.7%) | .23 |
| **Age (years)** | 30 – 39  40 – 49  50 – 60 | 141 (40.3%)  68 (19%)  30 (9%) | 121 (36.0%)  60 (18%)  33 (10%) | .28  .67  .66 |
| **Educational level** | No official education or primary school  Secondary school  Vocational education  Higher education | 19 (5%)  32 (9%)  72 (21%)  115 (32.9%) | 26 (8%)  32 (10%)  63 (19%)  93 (28%) | .29  .97  .61  .16 |
| **Generation** | First  Second | 85 (24%)  154 (44.0%) | 81 (24%)  133 (39.6%) | >.999  .27 |
| **Previous CC screening participation** | Every five years  Not every five years  Never | 121 (34.6%)  36 (10%)  82 (23%) | 107 (31.8%)  27 (8%)  80 (24%) | .50  .38  .98 |

CC: cervical cancer

**Table S2. Knowledge of CC screening among the control and intervention Moroccan-Dutch respondents, after the brochure (control) or brochure and CSEV (intervention)**

| **Characteristic** | | **Control group**  **N = 443** | **Intervention group**  **N = 435** | **P value** |
| --- | --- | --- | --- | --- |
|  |  | **Sufficient knowledge** | **Sufficient knowledge** |  |
| **Total group** | | 349 (78.8%) | 337 (77.5%) | .70 |
| **Age (years)** | 30 – 39  40 – 49  50 – 60 | 189 (42.7%)  130 (29.3%)  30 (7%) | 182 (41.8%)  127 (29.2%)  28 (6%) | .86  >.999  .95 |
| **Educational level** | No official education or primary school  Secondary school  Vocational education  Higher education | 20 (5%)  51 (12%)  83 (19%)  195 (44.0%) | 16 (4%)  45 (10%)  78 (18%)  197 (45.3%) | .65  .66  .83  .76 |
| **Generation** | First  Second | 144 (32.5%)  205 (46.3%) | 126 (29.0%)  211 (48.5%) | .29  .55 |
| **Previous CC screening participation** | Every five years  Not every five years  Never | 197 (44.5%)  44 (10%)  108 (24.4%) | 173 (39.8%)  55 (13%)  109 (25.1%) | .18  .25  .88 |

CC: cervical cancer

**Table S3. Attitude towards CC screening participation among the control and intervention Turkish-Dutch respondents, after the brochure (control) or brochure and CSEV (intervention)**

| **Characteristic** | | **Control group**  **N = 350** | **Intervention group**  **N = 336** | **P value** |
| --- | --- | --- | --- | --- |
|  |  | **Positive attitude** | **Positive attitude** |  |
| **Total group** | | 235 (67.1%) | 223 (66.4%) | .89 |
| **Age (years)** | 30 – 39  40 – 49  50 – 60 | 143 (40.9%)  62 (18%)  30 (9%) | 127 (37.8%)  62 (19%)  34 (10%) | .46  .88  .57 |
| **Educational level** | No official education or primary school  Secondary school  Vocational education  Higher education | 25 (7%)  30 (9%)  70 (20%)  110 (31.4%) | 24 (7%)  36 (11%)  60 (18%)  103 (30.7%) | >.999  .41  .54  .89 |
| **Generation** | First  Second | 94 (27%)  141 (40.3%) | 97 (29%)  126 (37.5%) | .62  .50 |
| **Previous CC screening participation** | Every five years  Not every five years  Never | 119 (34.0%)  36 (10%)  80 (23%) | 123 (36.6%)  36 (11%)  64 (19%) | .53  .95  .26 |

CC: cervical cancer

**Table S4. Attitude towards CC screening participation among the control and intervention Moroccan-Dutch respondents, after the brochure (control) or brochure and CSEV (intervention)**

| **Characteristic** | | **Control group**  **N = 443** | **Intervention group**  **N = 435** | **P value** |
| --- | --- | --- | --- | --- |
|  |  | **Positive attitude** | **Positive attitude** |  |
| **Total group** | | 303 (68.4%) | 323 (74.3%) | .07 |
| **Age (years)** | 30 – 39  40 – 49  50 – 60 | 155 (35.0%)  118 (26.6%)  30 (7%) | 173 (39.8%)  119 (27.4%)  31 (7%) | .16  .87  .94 |
| **Educational level** | No official education or primary school  Secondary school  Vocational education  Higher education | 22 (5%)  52 (12%)  77 (18%)  152 (34.3%) | 20 (5%)  49 (11%)  81 (19%)  172 (39.5%) | .92  .91  .70  .13 |
| **Generation** | First  Second | 134 (30.2%)  169 (38.1%) | 132 (30.3%)  191 (43.9%) | >.999  .10 |
| **Previous CC screening participation** | Every five years  Not every five years  Never | 180 (40.6%)  44 (10%)  79 (18%) | 172 (39.5%)  42 (10%)  109 (25.1%) | .79  .98  .01* |

CC: cervical cancer, * p < .05

**Table S5. Pre- and post-IDM in the control group, among Turkish-Dutch women**

| **Characteristic** | | **IDM after brochure** | | | P value |
| --- | --- | --- | --- | --- | --- |
|  |  | Yes, participation  **N = 152** | Yes, no participation  **N = 3** | No  **N = 195** |  |
| **Baseline IDM** | Yes, participation  Yes, no participation  No | 110 (72.4%)  0 (0%)  **42 (28%)** | 1 (33%)  1 (33%)  **1 (33%)** | 20 (10%)  3 (2%)  172 (88.2%) | <.001*** |

IDM: informed decision-making, * p < .05, ** p < .01, *** p < .001

**Table S6. Pre- and post-IDM in the intervention group, among Turkish-Dutch women**

| **Characteristic** | | **IDM after brochure and film** | | | P value |
| --- | --- | --- | --- | --- | --- |
|  |  | Yes, participation  **N = 140** | Yes, no participation  **N = 4** | No  **N = 192** |  |
| **Baseline IDM** | Yes, participation  Yes, no participation  No | 90 (64%)  0 (0%)  **50 (36%)** | 0 (0%)  1 (25%)  **3 (75%)** | 25 (13%)  0 (0%)  167 (87.0%) | <.001*** |

IDM: informed decision-making, * p < .05, ** p < .01, *** p < .001

**Table S7. Pre- and post-IDM in the control group, among Moroccan-Dutch women**

| **Characteristic** | | **IDM after brochure** | | | P value |
| --- | --- | --- | --- | --- | --- |
|  |  | Yes, participation  **N = 152** | Yes, no participation  **N = 3** | No  **N = 195** |  |
| **Baseline IDM** | Yes, participation  Yes, no participation  No | 172 (73.5%)  1 (0%)  **61 (26%)** | 0 (0%)  2 (50%)  **2 (50%)** | 19 (9%)  0 (0%)  186 (90.7%) | <0.001*** |

IDM: informed decision-making, * p < .05, ** p < .01, *** p < .001

**Table S8. Pre- and post-IDM in the intervention group, among Moroccan-Dutch women**

| **Characteristic** | | **IDM after brochure and film** | | | P value |
| --- | --- | --- | --- | --- | --- |
|  |  | Yes, participation  **N = 140** | Yes, no participation  **N = 4** | No  **N = 192** |  |
| **Baseline IDM** | Yes, participation  Yes, no participation  No | 175 (68.4%)  0 (0%)  **81 (32%)** | 0 (0%)  0 (0%)  0 (0%) | 14 (8%)  5 (3%)  160 (89.4%) | <0.001*** |

IDM: informed decision-making, * p < .05, ** p < .01, *** p < .001

**Table S9. Awareness, perceptions, and intention regarding self-sampling, after the brochure (control) or brochure and CSEV (intervention), among Turkish-Dutch women**

| **Characteristic** | | **Control group**  **N = 350** | **Intervention group**  **N = 336** | **P value** |
| --- | --- | --- | --- | --- |
| **Aware of the option of self-sampling** | Yes  No | 242 (69.1%)  108 (30.9%) | 254 (75.6%)  82 (24%) | .07 |
| **Ease of performance** | Yes  Do not know  No | 157 (44.9%)  140 (40.0%)  53 (15%) | 169 (50.3%)  120 (35.7%)  47 (14%) | .36 |
| **Painful** | Yes  Do not know  No | 56 (16%)  156 (44.6%)  138 (39.4%) | 51 (15%)  135 (40.2%)  150 (44.6%) | .38 |
| **Able to perform correctly** | Yes  Do not know  No | 152 (43.4%)  126 (36.0%)  72 (21%) | 154 (45.8%)  107 (31.8%)  75 (22%) | .51 |
| **Trust in the test result** | Yes  Do not know  No | 171 (48.9%)  137 (39.1%)  42 (12%) | 168 (50.0%)  120 (35.7%)  48 (14%) | .53 |
| **Intention to self-sample** | Yes  Do not know  No | 163 (46.6%)  59 (17%)  128 (36.6%) | 156 (46.4%)  72 (21%)  108 (32.1%) | .24 |

**Table S10. Awareness, perceptions, and intention regarding self-sampling, after the brochure (control) or brochure and CSEV (intervention), among Moroccan-Dutch women**

| **Characteristic** | | **Control group**  **N = 443** | **Intervention group**  **N = 435** | **P value** |
| --- | --- | --- | --- | --- |
| **Aware of the option of self-sampling** | Yes  No | 386 (87.1%)  57 (13%) | 390 (89.7%)  45 (10%) | .29 |
| **Ease of performance** | Yes  Do not know  No | 252 (56.9%)  135 (30.5%)  56 (13%) | 284 (65.3%)  106 (24.4%)  45 (10%) | .04* |
| **Painful** | Yes  Do not know  No | 82 (19%)  160 (36.1%)  201 (45.4%) | 59 (14%)  147 (33.8%)  229 (52.6%) | .048* |
| **Able to perform correctly** | Yes  Do not know  No | 255 (57.6%)  115 (26.0%)  73 (17%) | 272 (62.5%)  96 (22%)  67 (15%) | .30 |
| **Trust in the test result** | Yes  Do not know  No | 261 (58.9%)  128 (28.9%)  54 (12%) | 279 (64.1%)  110 (25.3%)  46 (11%) | .28 |
| **Intention to self-sample** | Yes  Do not know  No | 217 (49.0%)  76 (17%)  150 (33.9%) | 231 (53.1%)  79 (18%)  125 (28.7%) | .26 |

* p < 0.05

**References**

1. The GADM project. GADM version 2.8: a geographic database of global administrative areas. <https://gadm.org/>. 2018. Accessed 22 June 2019.
