# Supplementary material for: Evaluation of a Web-Based Culturally Sensitive Educational Video to Facilitate Informed Cervical Cancer Screening Decisions Among Turkish- and Moroccan-Dutch Women Aged 30 to 60 Years: Randomized Intervention Study
Source: J Med Internet Res. 2022 Oct 26;24(10):e35962. doi: 10.2196/35962 (PMC9647450; doi:10.2196/35962)
Supplement: Multimedia Appendix 2 [file jmir_v24i10e35962_app2.docx]

CONSORT-EHEALTH (V 1.6.1) -

Submission/Publication Form

The CONSORT-EHEALTH checklist is intended for authors of randomized trials evaluating web-based and Internet-based applications/interventions, including mobile interventions, electronic games (incl multiplayer games), social media, certain telehealth applications, and other interactive and/or networked electronic applications. Some of the items (e.g. all subitems under item 5 - description of the intervention) may also be applicable for other study designs.

The goal of the CONSORT EHEALTH checklist and guideline is to be

1. a guide for reporting for authors of RCTs,
2. to form a basis for appraisal of an ehealth trial (in terms of validity)

CONSORT-EHEALTH items/subitems are MANDATORY reporting items for studies published in the Journal of Medical Internet Research and other journals / scientific societies endorsing the checklist.

Items numbered 1., 2., 3., 4a., 4b etc are original CONSORT or CONSORT-NPT (non- pharmacologic treatment) items.

Items with Roman numerals (i., ii, iii, iv etc.) are CONSORT-EHEALTH extensions/clarifications.

As the CONSORT-EHEALTH checklist is still considered in a formative stage, we would ask that you also RATE ON A SCALE OF 1-5 how important/useful you feel each item is FOR THE PURPOSE OF THE CHECKLIST and reporting guideline (optional).

Mandatory reporting items are marked with a red *.

In the textboxes, either copy & paste the relevant sections from your manuscript into this form - please include any quotes from your manuscript in QUOTATION MARKS,

or answer directly by providing additional information not in the manuscript, or elaborating on why the item was not relevant for this study.

YOUR ANSWERS WILL BE PUBLISHED AS A SUPPLEMENTARY FILE TO YOUR PUBLICATION IN JMIR AND ARE CONSIDERED PART OF YOUR PUBLICATION (IF ACCEPTED).

Please fill in these questions diligently. Information will not be copyedited, so please use proper spelling and grammar, use correct capitalization, and avoid abbreviations.

DO NOT FORGET TO SAVE AS PDF _AND_ CLICK THE SUBMIT BUTTON SO YOUR ANSWERS ARE IN OUR DATABASE !!!

Citation Suggestion (if you append the pdf as Appendix we suggest to cite this paper in the caption):

Eysenbach G, CONSORT-EHEALTH Group

CONSORT-EHEALTH: Improving and Standardizing Evaluation Reports of Web-based and Mobile Health Interventions

J Med Internet Res 2011;13(4):e126
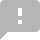
 URL: [http://www.jmir.org/2011/4/e126/](https://www.google.com/url?q=http%3A//www.jmir.org/2011/4/e126/&sa=D&source=editors&ust=1640271460186537&usg=AOvVaw1HBT6_oEAOAfNtYdWOLgUI)


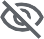

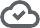


doi: 10.2196/jmir.1923

PMID: 22209829

[**norahamdiui@gmail.com**](mailto:norahamdiui@gmail.com) (niet gedeeld) [Ander account](https://accounts.google.com/AccountChooser?continue=https%3A//docs.google.com/forms/d/e/1FAIpQLSfZBSUp1bwOc_OimqcS64RdfIAFvmrTSkZQL2-3O8O9hrL5Sw/viewform%3Fhl%3Den_US%26formkey%3DdGlKd2Z2Q1lNSGQ0THl1azM5MS1aWWc6MA%26rm%3Dfull&service=wise)

Concept opgeslagen

*Vereist

Your name *

First Last

Nora Hamdiui

Primary Affiliation (short), City, Country *

University of Toronto, Toronto, Canada

National Coordination Centre for Communicab

Your e-mail address *

[abc@gmail.com](mailto:abc@gmail.com)

[nora.hamdiui@rivm.nl](mailto:nora.hamdiui@rivm.nl)

Title of your manuscript *

Provide the (draft) title of your manuscript.

Evaluation of a culturally sensitive educational video to facilitate informed cervical cancer screening decisions among Turkish- and Moroccan-Dutch women:

A randomised intervention study

Name of your App/Software/Intervention *

If there is a short and a long/alternate name, write the short name first and add the long name in brackets.

Culturally sensitive educational video

Evaluated Version (if any)

e.g. "V1", "Release 2017-03-01", "Version 2.0.27913"

Jouw antwoord

Language(s) *

What language is the intervention/app in? If multiple languages are available, separate by comma (e.g. "English, French")

Moroccan-Arabic, Moroccan-Berber, Turkish

URL of your Intervention Website or App

e.g. a direct link to the mobile app on app in appstore (itunes, Google Play), or URL of the website. If the intervention is a DVD or hardware, you can also link to an Amazon page.

https://[www.rivm.nl/bevolkingsonderzoek-baarmoederhalskanker/videos-marokkaans-turks](http://www.rivm.nl/bevolkingsonderzoek-baarmoederhalskanker/videos-marokkaans-turks)

URL of an image/screenshot (optional)

Jouw antwoord


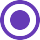

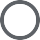

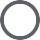

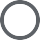

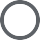


Accessibility *

Can an enduser access the intervention presently?

access is free and open

access only for special usergroups, not open

access is open to everyone, but requires payment/subscription/in-app purchases app/intervention no longer accessible

Anders:

Primary Medical Indication/Disease/Condition *

e.g. "Stress", "Diabetes", or define the target group in brackets after the condition, e.g. "Autism (Parents of children with)", "Alzheimers (Informal Caregivers of)"

Turkish- and Moroccan-Dutch women being 30

Primary Outcomes measured in trial *

comma-separated list of primary outcomes reported in the trial

Informed decision-making: knowledge, attitud

Secondary/other outcomes

Are there any other outcomes the intervention is expected to affect?

Reach, compared to the "usual care" (current information brochure)


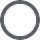

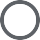

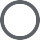

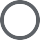

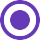

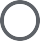


Recommended "Dose" *

What do the instructions for users say on how often the app should be used?

Approximately Daily Approximately Weekly Approximately Monthly Approximately Yearly "as needed"

Anders:


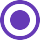

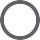

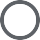

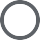

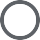

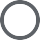

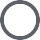

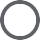

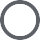

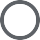

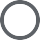

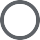


Approx. Percentage of Users (starters) still using the app as recommended after

3 months *

unknown / not evaluated 0-10%

11-20%

21-30%

31-40%

41-50%

51-60%

61-70%

71%-80%

81-90%

91-100%

Anders:


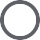

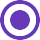

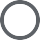

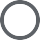

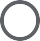

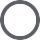


Overall, was the app/intervention effective? *

yes: all primary outcomes were significantly better in intervention group vs control

partly: SOME primary outcomes were significantly better in intervention group vs

control

no statistically significant difference between control and intervention

potentially harmful: control was significantly better than intervention in one or more

outcomes

inconclusive: more research is needed

Anders:


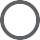

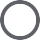

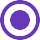

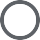

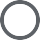

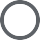

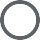


Article Preparation Status/Stage *

At which stage in your article preparation are you currently (at the time you fill in this form)

not submitted yet - in early draft status

not submitted yet - in late draft status, just before submission submitted to a journal but not reviewed yet

submitted to a journal and after receiving initial reviewer comments submitted to a journal and accepted, but not published yet published

Anders:


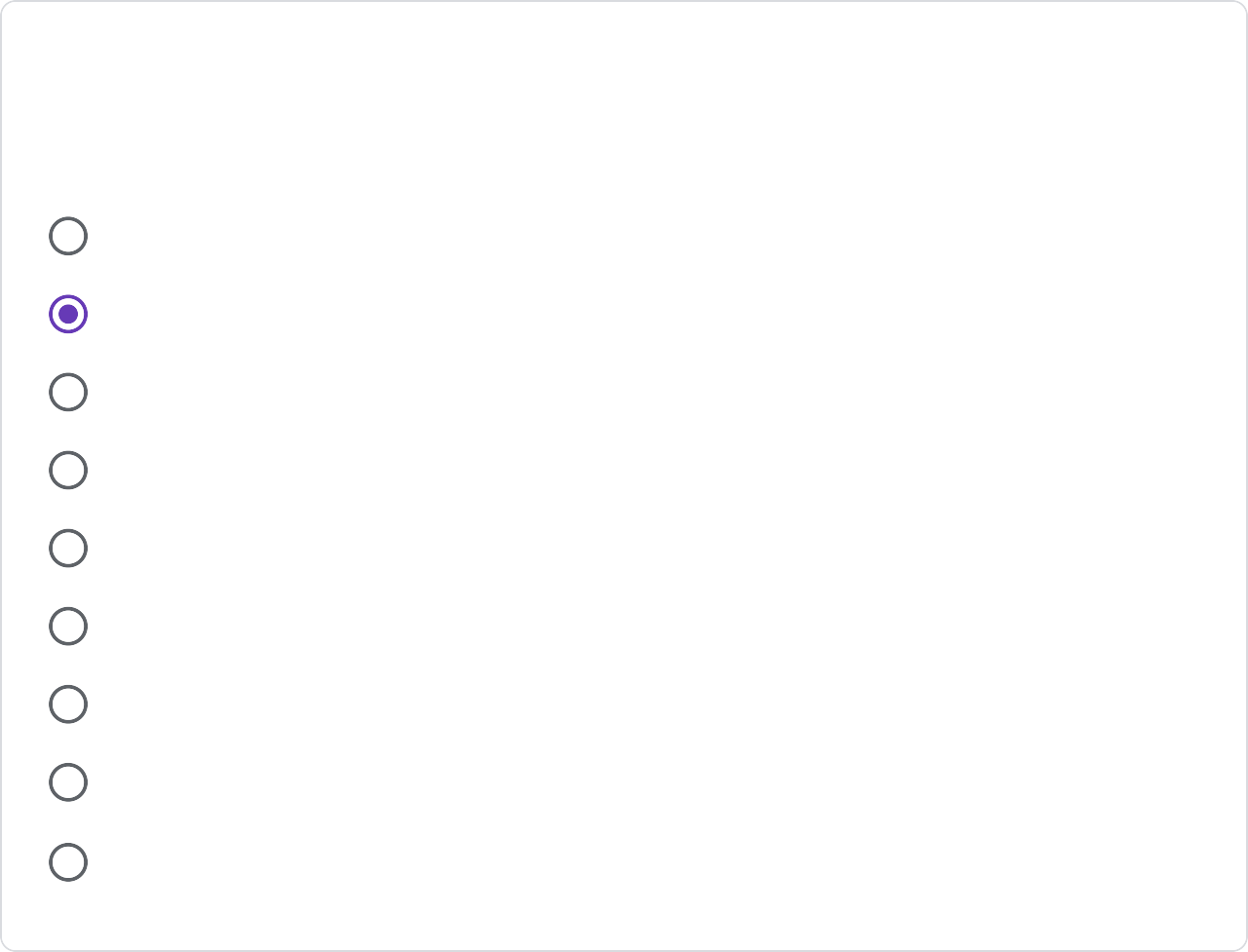


Journal *

If you already know where you will submit this paper (or if it is already submitted), please provide the journal name (if it is not JMIR, provide the journal name under "other")

not submitted yet / unclear where I will submit this Journal of Medical Internet Research (JMIR)

JMIR mHealth and UHealth JMIR Serious Games

JMIR Mental Health JMIR Public Health

JMIR Formative Research Other JMIR sister journal

Anders:


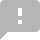


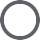

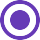


Is this a full powered effectiveness trial or a pilot/feasibility trial? *

Pilot/feasibility

Fully powered


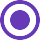

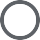


Manuscript tracking number *

If this is a JMIR submission, please provide the manuscript tracking number under "other" (The ms tracking number can be found in the submission acknowledgement email, or when you login as author in JMIR. If the paper is already published in JMIR, then the ms tracking number is the four-digit number at the end of the DOI, to be found at the bottom of each published article in JMIR)

no ms number (yet) / not (yet) submitted to / published in JMIR

Anders:

TITLE AND ABSTRACT

1a) TITLE: Identification as a randomized trial in the title


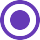

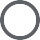


1a) Does your paper address CONSORT item 1a? *

I.e does the title contain the phrase "Randomized Controlled Trial"? (if not, explain the reason under "other")

yes

Anders:


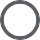

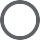

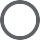

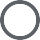

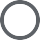


1a-i) Identify the mode of delivery in the title

Identify the mode of delivery. Preferably use “web-based” and/or “mobile” and/or “electronic game” in the title. Avoid ambiguous terms like “online”, “virtual”, “interactive”. Use “Internet-based” only if Intervention includes non-web-based Internet components (e.g. email), use “computer-based” or “electronic” only if offline products are used. Use “virtual” only in the context of “virtual reality” (3-D worlds). Use “online” only in the context of “online support groups”. Complement or substitute product names with broader terms for the class of products (such as “mobile” or “smart phone” instead of “iphone”), especially if the application runs on different platforms.

1

2

3

4

5

subitem not at all important

essential

Does your paper address subitem 1a-i? *

Copy and paste relevant sections from manuscript title (include quotes in quotation marks "like this" to indicate direct quotes from your manuscript), or elaborate on this item by providing additional information not in the ms, or briefly explain why the item is not applicable/relevant for your study

"Evaluation of a web-based culturally sensitive educational video to facilitate informed cervical cancer screening decisions among Turkish- and Moroccan-Dutch women aged 30 to

60 years: A randomized intervention study"


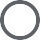

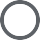

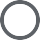

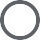

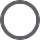


1a-ii) Non-web-based components or important co-interventions in title

Mention non-web-based components or important co-interventions in title, if any (e.g., “with telephone support”).

1

2

3

4

5

subitem not at all important

essential

Does your paper address subitem 1a-ii?

Copy and paste relevant sections from manuscript title (include quotes in quotation marks "like this" to indicate direct quotes from your manuscript), or elaborate on this item by providing additional information not in the ms, or briefly explain why the item is not applicable/relevant for your study

Jouw antwoord


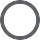

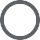

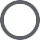

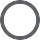

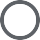


1a-iii) Primary condition or target group in the title

Mention primary condition or target group in the title, if any (e.g., “for children with Type I Diabetes”) Example: A Web-based and Mobile Intervention with Telephone Support for Children with Type I Diabetes: Randomized Controlled Trial

1

2

3

4

5

subitem not at all important

essential

Does your paper address subitem 1a-iii? *

Copy and paste relevant sections from manuscript title (include quotes in quotation marks "like this" to indicate direct quotes from your manuscript), or elaborate on this item by providing additional information not in the ms, or briefly explain why the item is not applicable/relevant for your study

"Evaluation of a web-based culturally sensitive educational video to facilitate informed cervical cancer screening decisions among Turkish- and Moroccan-Dutch women aged 30 to

60 years: A randomized intervention study"

1b) ABSTRACT: Structured summary of trial design, methods, results, and conclusions

NPT extension: Description of experimental treatment, comparator, care providers, centers, and blinding

status.


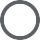

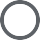

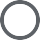

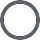

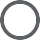


1b-i) Key features/functionalities/components of the intervention and comparator in the METHODS section of the ABSTRACT

Mention key features/functionalities/components of the intervention and comparator in the abstract. If possible, also mention theories and principles used for designing the site. Keep in mind the needs of systematic reviewers and indexers by including important synonyms. (Note: Only report in the abstract what the main paper is reporting. If this information is missing from the main body of text, consider adding it)

1

2

3

4

5

subitem not at all important

essential

Does your paper address subitem 1b-i? *

Copy and paste relevant sections from the manuscript abstract (include quotes in quotation marks "like this" to indicate direct quotes from your manuscript), or elaborate on this item by providing additional information not in the ms, or briefly explain why the item is not applicable/relevant for your study

"Respondents were randomly assigned to the control (current information brochure), or

intervention condition (brochure and CSEV)."


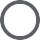

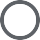

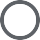

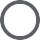

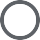


1b-ii) Level of human involvement in the METHODS section of the ABSTRACT

Clarify the level of human involvement in the abstract, e.g., use phrases like “fully automated” vs. “therapist/nurse/care provider/physician-assisted” (mention number and expertise of providers involved, if any). (Note: Only report in the abstract what the main paper is reporting. If this information is missing from the main body of text, consider adding it)

1

2

3

4

5

subitem not at all important

essential

Does your paper address subitem 1b-ii?

Copy and paste relevant sections from the manuscript abstract (include quotes in quotation marks "like this" to indicate direct quotes from your manuscript), or elaborate on this item by providing additional information not in the ms, or briefly explain why the item is not applicable/relevant for your study

Jouw antwoord


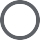

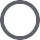

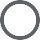

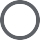

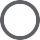


1b-iii) Open vs. closed, web-based (self-assessment) vs. face-to-face assessments in the METHODS section of the ABSTRACT

Mention how participants were recruited (online vs. offline), e.g., from an open access website or from a clinic or a closed online user group (closed usergroup trial), and clarify if this was a purely web-based trial, or there were face-to-face components (as part of the intervention or for assessment). Clearly say if outcomes were self-assessed through questionnaires (as common in web-based trials). Note: In traditional offline trials, an open trial (open-label trial) is a type of clinical trial in which both the researchers and participants know which treatment is being administered. To avoid confusion, use “blinded” or “unblinded” to indicated the level of blinding instead of “open”, as “open” in web-based trials usually refers to “open access” (i.e. participants can self-enrol). (Note: Only report in the abstract what the main paper is reporting. If this information is missing from the main body of text, consider adding it)

1

2

3

4

5

subitem not at all important

essential

Does your paper address subitem 1b-iii?

Copy and paste relevant sections from the manuscript abstract (include quotes in quotation marks "like this" to indicate direct quotes from your manuscript), or elaborate on this item by providing additional information not in the ms, or briefly explain why the item is not applicable/relevant for your study

"Initial respondents were recruited via several social media platforms, and invited to complete an online questionnaire. Following respondent-driven sampling, respondents were asked to recruit a number of peers from their social network to complete the same

questionnaire."


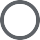

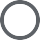

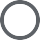

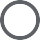

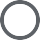


1b-iv) RESULTS section in abstract must contain use data

Report number of participants enrolled/assessed in each group, the use/uptake of the intervention (e.g., attrition/adherence metrics, use over time, number of logins etc.), in addition to primary/secondary outcomes. (Note: Only report in the abstract what the main paper is reporting. If this information is missing from the main body of text, consider adding it)

1

2

3

4

5

subitem not at all important

essential

Does your paper address subitem 1b-iv?

Copy and paste relevant sections from the manuscript abstract (include quotes in quotation marks "like this" to indicate direct quotes from your manuscript), or elaborate on this item by providing additional information not in the ms, or briefly explain why the item is not applicable/relevant for your study

"The final sample included 686 Turkish- and 878 Moroccan-Dutch women. Of this sample, 793 were randomized to the control group (350 Turkish and 443 Moroccan) and 771 to the intervention group (336 Turkish and 435 Moroccan). Among Turkish-Dutch women, 33.1% of the control respondents and 40.5% of the intervention respondents consulted the brochure (not statistically significant). Among Moroccan-Dutch women, these percentages were 28.2% and 37.9%, respectively (P = 0.003). Of all intervention respondents, 96.1% (Turkish) and 84.4% (Moroccan) consulted the CSEV. The CSEV resulted in more positive screening attitudes among Moroccan-Dutch women, in comparison to the brochure (74.3% versus

68.4%, P = 0.07)."


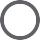

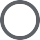

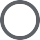

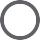

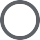


1b-v) CONCLUSIONS/DISCUSSION in abstract for negative trials

Conclusions/Discussions in abstract for negative trials: Discuss the primary outcome - if the trial is negative (primary outcome not changed), and the intervention was not used, discuss whether negative results are attributable to lack of uptake and discuss reasons. (Note: Only report in the abstract what the main paper is reporting. If this information is missing from the main body of text, consider adding it)

1

2

3

4

5

subitem not at all important

essential

Does your paper address subitem 1b-v?

Copy and paste relevant sections from the manuscript abstract (include quotes in quotation marks "like this" to indicate direct quotes from your manuscript), or elaborate on this item by providing additional information not in the ms, or briefly explain why the item is not applicable/relevant for your study

Jouw antwoord

INTRODUCTION

2a) In INTRODUCTION: Scientific background and explanation of rationale


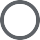

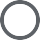

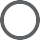

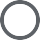

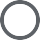


2a-i) Problem and the type of system/solution

Describe the problem and the type of system/solution that is object of the study: intended as stand-alone intervention vs. incorporated in broader health care program? Intended for a particular patient population? Goals of the intervention, e.g., being more cost-effective to other interventions, replace or complement other solutions? (Note: Details about the intervention are provided in “Methods” under 5)

1

2

3

4

5

subitem not at all important

essential

Does your paper address subitem 2a-i? *

Copy and paste relevant sections from the manuscript (include quotes in quotation marks "like this" to indicate direct quotes from your manuscript), or elaborate on this item by providing additional information not in the ms, or briefly explain why the item is not applicable/relevant for your study

"In this study, we evaluated the effect of this CSEV on IDM regarding CC screening participation among Turkish- and Moroccan-Dutch women. We hypothesized that adding a CSEV to the current Dutch information brochure would increase the IDM on participation in

CC screening of these women."


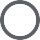

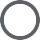

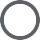

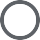

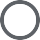


2a-ii) Scientific background, rationale: What is known about the (type of) system

Scientific background, rationale: What is known about the (type of) system that is the object of the study (be sure to discuss the use of similar systems for other conditions/diagnoses, if appropiate), motivation for the study, i.e. what are the reasons for and what is the context for this specific study, from which stakeholder viewpoint is the study performed, potential impact of findings [2]. Briefly justify the choice of the comparator.

1

2

3

4

5

subitem not at all important

essential

# Does your paper address subitem 2a-ii? *

Copy and paste relevant sections from the manuscript (include quotes in quotation marks "like this" to indicate direct quotes from your manuscript), or elaborate on this item by providing additional information not in the ms, or briefly explain why the item is not applicable/relevant for your study

"In decision-making, Turkish- and Moroccan-Dutch women not only take factual medical information into account, but also consider practical, emotional, cultural, and religious aspects prior to deciding to screen or not [5]. However, the current invitation letter and information brochure contain predominantly factual medical information. Turkish- and Moroccan-Dutch women often indicated to not (thoroughly) read the invitation letter and brochure, or simply being unable to understand these materials due to a lack of a good command of the Dutch language [5]. These women were also shown to make less use of printed media and more of audio-visual media [7]. As a Culturally Competent Educational Film, developed with peer educators, was successful in improving IDM for prenatal screening among pregnant ethnic minority women, we considered this beneficial for IDM in CC screening participation as well [8]. Thus, we developed a Culturally Sensitive Educational Video (CSEV) incorporating more affective information and distributed it via respondent- driven sampling (RDS)."

2b) In INTRODUCTION: Specific objectives or hypotheses

Does your paper address CONSORT subitem 2b? *

Copy and paste relevant sections from the manuscript (include quotes in quotation marks "like this" to indicate direct quotes from your manuscript), or elaborate on this item by providing additional information not in the ms, or briefly explain why the item is not applicable/relevant for your study

"In this study, we evaluated the effect of this CSEV on IDM regarding CC screening participation among Turkish- and Moroccan-Dutch women. We hypothesized that adding a CSEV to the current Dutch information brochure would increase the IDM on participation in

CC screening of these women."

METHODS

3a) Description of trial design (such as parallel, factorial) including allocation ratio

Does your paper address CONSORT subitem 3a? *

Copy and paste relevant sections from the manuscript (include quotes in quotation marks "like this" to indicate direct quotes from your manuscript), or elaborate on this item by providing additional information not in the ms, or briefly explain why the item is not applicable/relevant for your study

"Between 23 November 2020 and 6 August 2021, a randomized intervention study was

conducted with a control and an intervention group."

3b) Important changes to methods after trial commencement (such as eligibility criteria), with reasons

Does your paper address CONSORT subitem 3b? *

Copy and paste relevant sections from the manuscript (include quotes in quotation marks "like this" to indicate direct quotes from your manuscript), or elaborate on this item by providing additional information not in the ms, or briefly explain why the item is not applicable/relevant for your study

There were no important changes to methods after trial commencement.


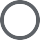

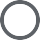

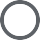


3b-i) Bug fixes, Downtimes, Content Changes

Bug fixes, Downtimes, Content Changes: ehealth systems are often dynamic systems. A description of changes to methods therefore also includes important changes made on the intervention or comparator during the trial (e.g., major bug fixes or changes in the functionality or content) (5-iii) and other “unexpected events” that may have influenced study design such as staff changes, system failures/downtimes, etc. [2].

1

2

3

4

5

subitem not at all important

essential

Does your paper address subitem 3b-i?

Copy and paste relevant sections from the manuscript (include quotes in quotation marks "like this" to indicate direct quotes from your manuscript), or elaborate on this item by providing additional information not in the ms, or briefly explain why the item is not applicable/relevant for your study

Jouw antwoord

4a) Eligibility criteria for participants

Does your paper address CONSORT subitem 4a? *

Copy and paste relevant sections from the manuscript (include quotes in quotation marks "like this" to indicate direct quotes from your manuscript), or elaborate on this item by providing additional information not in the ms, or briefly explain why the item is not applicable/relevant for your study

"Inclusion criteria for respondents were: 1) women being 30 to 60 years old, and 2) being born in Turkey or Morocco, and having at least one parent born in Turkey or Morocco (first- generation immigrants), or being born in the Netherlands, and having at least one parent

born in Turkey or Morocco (second-generation immigrants), and 3) living in the Netherlands."

4a-i) Computer / Internet literacy

Computer / Internet literacy is often an implicit “de facto” eligibility criterion - this should be explicitly clarified.

1

2

3

4

5

subitem not at all important

essential

Does your paper address subitem 4a-i?

Copy and paste relevant sections from the manuscript (include quotes in quotation marks "like this" to indicate direct quotes from your manuscript), or elaborate on this item by providing additional information not in the ms, or briefly explain why the item is not applicable/relevant for your study

Jouw antwoord

4a-ii) Open vs. closed, web-based vs. face-to-face assessments:

Open vs. closed, web-based vs. face-to-face assessments: Mention how participants were recruited (online vs. offline), e.g., from an open access website or from a clinic, and clarify if this was a purely web- based trial, or there were face-to-face components (as part of the intervention or for assessment), i.e., to what degree got the study team to know the participant. In online-only trials, clarify if participants were quasi-anonymous and whether having multiple identities was possible or whether technical or logistical measures (e.g., cookies, email confirmation, phone calls) were used to detect/prevent these.

1

2

3

4

5

subitem not at all important

essential

# Does your paper address subitem 4a-ii? *

Copy and paste relevant sections from the manuscript (include quotes in quotation marks "like this" to indicate direct quotes from your manuscript), or elaborate on this item by providing additional information not in the ms, or briefly explain why the item is not applicable/relevant for your study

"Seeds were recruited via several social media platforms, such as (1) public and private women’s groups on Facebook, (2) the LinkedIn pages of the involved researchers, (3) the foundation called the Association Moroccan Doctors Netherlands (AMAN), and (4) the participating video producer Zouka Media. We also (5) contacted several influencers on Instagram with many Turkish- and/or Moroccan-Dutch female followers and asked them to share the questionnaire via their story or bio. Throughout the study, we used paper- and web-based flyers and an online infographic to promote and share the link to the questionnaire. The flyers and infographic were spread among offline community organizations, foundations, and mosques, as well as online platforms, such as LinkedIn and Facebook.

After completion of the questionnaire, respondents were asked to invite - through WhatsApp, e-mail, platforms as Instagram, and/or SMS - a maximum of 20 women from their social network to complete the same questionnaire. In case of e-mail, reminders were sent to complete and/or forward the questionnaire, and to encourage respondents to remind their peers to complete the questionnaire (after one week of no participation of at least one peer)."

4a-iii) Information giving during recruitment

Information given during recruitment. Specify how participants were briefed for recruitment and in the informed consent procedures (e.g., publish the informed consent documentation as appendix, see also item X26), as this information may have an effect on user self-selection, user expectation and may also bias results.

1

2

3

4

5

subitem not at all important

essential

Does your paper address subitem 4a-iii?

Copy and paste relevant sections from the manuscript (include quotes in quotation marks "like this" to indicate direct quotes from your manuscript), or elaborate on this item by providing additional information not in the ms, or briefly explain why the item is not applicable/relevant for your study

"Respondents were informed about the study (but did not know there was a control and an

intervention group), and asked to give their digital informed consent."

4b) Settings and locations where the data were collected

Does your paper address CONSORT subitem 4b? *

Copy and paste relevant sections from the manuscript (include quotes in quotation marks "like this" to indicate direct quotes from your manuscript), or elaborate on this item by providing additional information not in the ms, or briefly explain why the item is not applicable/relevant for your study

"RDS starts with a convenience, ideally diverse, sample of members of the population, which are called seeds [11]. Seeds are asked to complete a questionnaire and to recruit a number of their peers to complete the same questionnaire. Successfully recruited peers are then also asked to recruit a number of peers. This recruitment was continued until the calculated sample size was reached. Unique tokens were used to follow who recruited whom and draw recruitment trees. Each new respondent was randomly assigned to either the control or

intervention condition (i.e. individual level randomization) (see Figure 1)."

4b-i) Report if outcomes were (self-)assessed through online questionnaires

Clearly report if outcomes were (self-)assessed through online questionnaires (as common in web-based trials) or otherwise.

1

2

3

4

5

subitem not at all important

essential

Does your paper address subitem 4b-i? *

Copy and paste relevant sections from the manuscript (include quotes in quotation marks "like this" to indicate direct quotes from your manuscript), or elaborate on this item by providing additional information not in the ms, or briefly explain why the item is not applicable/relevant for your study

"Respondents were asked to complete an online questionnaire, in which questions on IDM were asked before and after the control or intervention condition. The control group was asked to read the information brochure regarding the screening program that is currently sent with the screening invitation. The intervention group was asked to read the same brochure and watch the CSEV. This request was shown on one webpage. By clicking ‘Next’, they first received the brochure, and subsequently on the next page, the CSEV was

displayed."

4b-ii) Report how institutional affiliations are displayed

Report how institutional affiliations are displayed to potential participants [on ehealth media], as affiliations with prestigious hospitals or universities may affect volunteer rates, use, and reactions with regards to an intervention.(Not a required item – describe only if this may bias results)

1

2

3

4

5

subitem not at all important

essential

Does your paper address subitem 4b-ii?

Copy and paste relevant sections from the manuscript (include quotes in quotation marks "like this" to indicate direct quotes from your manuscript), or elaborate on this item by providing additional information not in the ms, or briefly explain why the item is not applicable/relevant for your study

Jouw antwoord

5) The interventions for each group with sufficient details to allow replication, including how and when they were actually administered

5-i) Mention names, credential, affiliations of the developers, sponsors, and owners

Mention names, credential, affiliations of the developers, sponsors, and owners [6] (if authors/evaluators are owners or developer of the software, this needs to be declared in a “Conflict of interest” section or mentioned elsewhere in the manuscript).

1

2

3

4

5

subitem not at all important

essential

Does your paper address subitem 5-i?

Copy and paste relevant sections from the manuscript (include quotes in quotation marks "like this" to indicate direct quotes from your manuscript), or elaborate on this item by providing additional information not in the ms, or briefly explain why the item is not applicable/relevant for your study

"We developed three Culturally Sensitive Educational Videos (CSEVs) in collaboration with

the video producer, and eight Turkish- and Moroccan-Dutch peer educators and actresses."

5-ii) Describe the history/development process

Describe the history/development process of the application and previous formative evaluations (e.g., focus groups, usability testing), as these will have an impact on adoption/use rates and help with interpreting results.

1

2

3

4

5

subitem not at all important

essential

Does your paper address subitem 5-ii?

Copy and paste relevant sections from the manuscript (include quotes in quotation marks "like this" to indicate direct quotes from your manuscript), or elaborate on this item by providing additional information not in the ms, or briefly explain why the item is not applicable/relevant for your study

"Finally, we based the content of the CSEVs on our earlier conducted focus groups among

offline-recruited Turkish- and Moroccan-Dutch women [5]."

5-iii) Revisions and updating

Revisions and updating. Clearly mention the date and/or version number of the application/intervention (and comparator, if applicable) evaluated, or describe whether the intervention underwent major changes during the evaluation process, or whether the development and/or content was “frozen” during the trial. Describe dynamic components such as news feeds or changing content which may have an impact on the replicability of the intervention (for unexpected events see item 3b).

1

2

3

4

5

subitem not at all important

essential

Does your paper address subitem 5-iii?

Copy and paste relevant sections from the manuscript (include quotes in quotation marks "like this" to indicate direct quotes from your manuscript), or elaborate on this item by providing additional information not in the ms, or briefly explain why the item is not applicable/relevant for your study

Jouw antwoord

5-iv) Quality assurance methods

Provide information on quality assurance methods to ensure accuracy and quality of information provided [1], if applicable.

1

2

3

4

5

subitem not at all important

essential

Does your paper address subitem 5-iv?

Copy and paste relevant sections from the manuscript (include quotes in quotation marks "like this" to indicate direct quotes from your manuscript), or elaborate on this item by providing additional information not in the ms, or briefly explain why the item is not applicable/relevant for your study

Jouw antwoord

5-v) Ensure replicability by publishing the source code, and/or providing screenshots/screen-capture video, and/or providing flowcharts of the algorithms used

Ensure replicability by publishing the source code, and/or providing screenshots/screen-capture video, and/or providing flowcharts of the algorithms used. Replicability (i.e., other researchers should in principle be able to replicate the study) is a hallmark of scientific reporting.

1

2

3

4

5

subitem not at all important

essential

Does your paper address subitem 5-v?

Copy and paste relevant sections from the manuscript (include quotes in quotation marks "like this" to indicate direct quotes from your manuscript), or elaborate on this item by providing additional information not in the ms, or briefly explain why the item is not applicable/relevant for your study

Jouw antwoord

5-vi) Digital preservation

Digital preservation: Provide the URL of the application, but as the intervention is likely to change or disappear over the course of the years; also make sure the intervention is archived (Internet Archive, [webcitation.org](https://www.google.com/url?q=http%3A//webcitation.org&sa=D&source=editors&ust=1640271460242654&usg=AOvVaw2Itjbv5Tesa_y-dSQ9yN6V), and/or publishing the source code or screenshots/videos alongside the article). As pages behind login screens cannot be archived, consider creating demo pages which are accessible without login.

1

2

3

4

5

subitem not at all important

essential

Does your paper address subitem 5-vi?

Copy and paste relevant sections from the manuscript (include quotes in quotation marks "like this" to indicate direct quotes from your manuscript), or elaborate on this item by providing additional information not in the ms, or briefly explain why the item is not applicable/relevant for your study

"All CSEVs are available via the official webpage of the Dutch National Institute for Public

Health and the Environment (RIVM) [15]."

5-vii) Access

Access: Describe how participants accessed the application, in what setting/context, if they had to pay (or were paid) or not, whether they had to be a member of specific group. If known, describe how participants obtained “access to the platform and Internet” [1]. To ensure access for editors/reviewers/readers, consider to provide a “backdoor” login account or demo mode for reviewers/readers to explore the application (also important for archiving purposes, see vi).

1

2

3

4

5

subitem not at all important

essential

Does your paper address subitem 5-vii? *

Copy and paste relevant sections from the manuscript (include quotes in quotation marks "like this" to indicate direct quotes from your manuscript), or elaborate on this item by providing additional information not in the ms, or briefly explain why the item is not applicable/relevant for your study

"Respondents were asked to complete an online questionnaire, in which questions on IDM were asked before and after the control or intervention condition. The control group was asked to read the information brochure regarding the screening program that is currently sent with the screening invitation. The intervention group was asked to read the same brochure and watch the CSEV. This request was shown on one webpage. By clicking ‘Next’, they first received the brochure, and subsequently on the next page, the CSEV was

displayed."

5-viii) Mode of delivery, features/functionalities/components of the intervention and comparator, and the theoretical framework

Describe mode of delivery, features/functionalities/components of the intervention and comparator, and the theoretical framework [6] used to design them (instructional strategy [1], behaviour change techniques, persuasive features, etc., see e.g., [7, 8] for terminology). This includes an in-depth description of the content (including where it is coming from and who developed it) [1],” whether [and how] it is tailored to individual circumstances and allows users to track their progress and receive feedback” [6]. This also includes a description of communication delivery channels and – if computer- mediated communication is a component – whether communication was synchronous or asynchronous [6]. It also includes information on presentation strategies [1], including page design principles, average amount of text on pages, presence of hyperlinks to other resources, etc. [1].

1

2

3

4

5

subitem not at all important

essential

# Does your paper address subitem 5-viii? *

Copy and paste relevant sections from the manuscript (include quotes in quotation marks "like this" to indicate direct quotes from your manuscript), or elaborate on this item by providing additional information not in the ms, or briefly explain why the item is not applicable/relevant for your study

"We developed three Culturally Sensitive Educational Videos (CSEVs) in collaboration with the video producer, and eight Turkish- and Moroccan-Dutch peer educators and actresses. Since all respondents received the brochure containing cognitive information on CC screening, we focused the video on affective information related to CC screening (i.e. experiences and fears). Turkish- and Moroccan-Dutch women especially need information on practical, emotional, cultural, and religious aspects of CC screening [5]. Therefore, the CSEVs emphasized three themes regarding clinician-based sampling, and ensured balanced content in terms of possible benefits and adverse effects. The themes included “more assurance regarding health and the ability to prevent treatment, surgery, or death, and because of this, being there for their children”, “according to the Islam, a woman should take good care of her health”, and “anxiety, shame, and privacy”. For self-sampling, two themes were included, namely “it is easy and not painful to perform self-sampling” and “trust in themselves to correctly perform self-sampling and trust in the test result”. The CSEV was available in Turkish, Moroccan-Arabic, and Moroccan-Berber (all with Dutch subtitles).

Moroccan-Dutch respondents could choose between a Moroccan-Arabic-spoken or Moroccan-Berber-spoken video."

5-ix) Describe use parameters

Describe use parameters (e.g., intended “doses” and optimal timing for use). Clarify what instructions or recommendations were given to the user, e.g., regarding timing, frequency, heaviness of use, if any, or was the intervention used ad libitum.

1

2

3

4

5

subitem not at all important

essential

Does your paper address subitem 5-ix?

Copy and paste relevant sections from the manuscript (include quotes in quotation marks "like this" to indicate direct quotes from your manuscript), or elaborate on this item by providing additional information not in the ms, or briefly explain why the item is not applicable/relevant for your study

"Respondents were asked to complete an online questionnaire, in which questions on IDM were asked before and after the control or intervention condition. The control group was asked to read the information brochure regarding the screening program that is currently sent with the screening invitation. The intervention group was asked to read the same brochure and watch the CSEV. This request was shown on one webpage. By clicking ‘Next’, they first received the brochure, and subsequently on the next page, the CSEV was

displayed."

5-x) Clarify the level of human involvement

Clarify the level of human involvement (care providers or health professionals, also technical assistance) in the e-intervention or as co-intervention (detail number and expertise of professionals involved, if any, as well as “type of assistance offered, the timing and frequency of the support, how it is initiated, and the medium by which the assistance is delivered”. It may be necessary to distinguish between the level of human involvement required for the trial, and the level of human involvement required for a routine application outside of a RCT setting (discuss under item 21 – generalizability).

1

2

3

4

5

subitem not at all important

essential

Does your paper address subitem 5-x?

Copy and paste relevant sections from the manuscript (include quotes in quotation marks "like this" to indicate direct quotes from your manuscript), or elaborate on this item by providing additional information not in the ms, or briefly explain why the item is not applicable/relevant for your study

Jouw antwoord

5-xi) Report any prompts/reminders used

Report any prompts/reminders used: Clarify if there were prompts (letters, emails, phone calls, SMS) to use the application, what triggered them, frequency etc. It may be necessary to distinguish between the level of prompts/reminders required for the trial, and the level of prompts/reminders for a routine application outside of a RCT setting (discuss under item 21 – generalizability).

1

2

3

4

5

subitem not at all important

essential

Does your paper address subitem 5-xi? *

Copy and paste relevant sections from the manuscript (include quotes in quotation marks "like this" to indicate direct quotes from your manuscript), or elaborate on this item by providing additional information not in the ms, or briefly explain why the item is not applicable/relevant for your study

"After completion of the questionnaire, respondents were asked to invite - through WhatsApp, e-mail, platforms as Instagram, and/or SMS - a maximum of 20 women from their social network to complete the same questionnaire. In case of e-mail, reminders were sent to complete and/or forward the questionnaire, and to encourage respondents to remind their peers to complete the questionnaire (after one week of no participation of at least one

peer)."

5-xii) Describe any co-interventions (incl. training/support)

Describe any co-interventions (incl. training/support): Clearly state any interventions that are provided in addition to the targeted eHealth intervention, as ehealth intervention may not be designed as stand-alone intervention. This includes training sessions and support [1]. It may be necessary to distinguish between the level of training required for the trial, and the level of training for a routine application outside of a RCT setting (discuss under item 21 – generalizability.

1

2

3

4

5

subitem not at all important

essential

Does your paper address subitem 5-xii? *

Copy and paste relevant sections from the manuscript (include quotes in quotation marks "like this" to indicate direct quotes from your manuscript), or elaborate on this item by providing additional information not in the ms, or briefly explain why the item is not applicable/relevant for your study

"Respondents were asked to complete an online questionnaire, in which questions on IDM were asked before and after the control or intervention condition. The control group was asked to read the information brochure regarding the screening program that is currently sent with the screening invitation. The intervention group was asked to read the same brochure and watch the CSEV. This request was shown on one webpage. By clicking ‘Next’, they first received the brochure, and subsequently on the next page, the CSEV was

displayed."

6a) Completely defined pre-specified primary and secondary outcome measures, including how and when they were assessed

6a-i) Online questionnaires: describe if they were validated for online use and apply CHERRIES items to describe how the questionnaires were designed/deployed

If outcomes were obtained through online questionnaires, describe if they were validated for online use and apply CHERRIES items to describe how the questionnaires were designed/deployed [9].

1

2

3

4

5

subitem not at all important

essential

# Does your paper address CONSORT subitem 6a? *

Copy and paste relevant sections from the manuscript (include quotes in quotation marks "like this" to indicate direct quotes from your manuscript), or elaborate on this item by providing additional information not in the ms, or briefly explain why the item is not applicable/relevant for your study

"We developed a questionnaire for measuring informed decision-making (IDM) based on the Rational Decision Model, that supposes that decision-making is based on a proper understanding of the potential benefits and adverse effects of cancer screening (decision- relevant knowledge) in the context of their personal situation and preferences (attitude) [12]. The questionnaire contained 52 questions regarding socio-demographic characteristics, previous CC screening participation, knowledge regarding CC screening, attitude towards CC screening, and intention to participate in the next CC screening round. Questions regarding knowledge, attitude, and intention were asked for clinician-based sampling, whereas for self- sampling, we included questions on awareness, perceptions, and intention. The rationale for this difference was that the self-sampling method was only introduced in 2017, which means that not every woman is aware of its existence. Therefore, instead of assessing knowledge and attitude, we questioned their awareness and perceptions on self-sampling.

Knowledge regarding CC screening was measured using three questions about the subsequent steps following a test result and the possibility of false-positive test results, with a score ranging from zero to four. Attitude towards CC screening was measured using ten questions, with a score ranging from zero to ten. These scores were transformed to zero to 100 scores to facilitate interpretation, following an earlier study of Korfage et al. [13]. In agreement with Van den Berg et al. [14] and Korfage et al. [13], we classified scores in the range of 45 to 55 as a neutral attitude. Scores below 45 were classified as a negative attitude, and scores above 55 as a positive attitude. Intention was measured by asking respondents whether they intended to participate in the next CC screening round. All questions regarding attitude and intention had three response options, namely ‘Yes’, ‘I do not know’, and ‘No’.

Following earlier research, we combined knowledge, attitude, and intention to calculate IDM (yes/no) [4,8]. An informed decision was defined as having adequate knowledge (total score

≥ 3.0), either a positive attitude (total score > 55.0), and a positive intention, or a negative attitude (total score < 45.0), and a negative intention. All other combinations were defined as an uninformed decision."

Does your paper address subitem 6a-i?

Copy and paste relevant sections from manuscript text

Jouw antwoord

6a-ii) Describe whether and how “use” (including intensity of use/dosage) was defined/measured/monitored

Describe whether and how “use” (including intensity of use/dosage) was defined/measured/monitored (logins, logfile analysis, etc.). Use/adoption metrics are important process outcomes that should be reported in any ehealth trial.

1

2

3

4

5

subitem not at all important

essential

Does your paper address subitem 6a-ii?

Copy and paste relevant sections from manuscript text

"Through automatic registration by the questionnaire software, we measured whether and

how long respondents consulted the brochure (in both the control and intervention group), and whether the intervention group actually watched the CSEV."

6a-iii) Describe whether, how, and when qualitative feedback from participants was obtained

Describe whether, how, and when qualitative feedback from participants was obtained (e.g., through emails, feedback forms, interviews, focus groups).

1

2

3

4

5

subitem not at all important

essential

Does your paper address subitem 6a-iii?

Copy and paste relevant sections from manuscript text

"To verify whether the CSEVs were understandable and culturally appropriate, online discussions were held between experts on language, communication, culture, and CC (screening). The CSEVs were also pilot tested in a small sample of Turkish- and Moroccan- Dutch women to verify whether the feasibility, content, and lay-out matched their needs and

requirements."

6b) Any changes to trial outcomes after the trial commenced, with reasons

Does your paper address CONSORT subitem 6b? *

Copy and paste relevant sections from the manuscript (include quotes in quotation marks "like this" to indicate direct quotes from your manuscript), or elaborate on this item by providing additional information not in the ms, or briefly explain why the item is not applicable/relevant for your study

There were no changes to trial outcomes after the trial commenced.

7a) How sample size was determined

NPT: When applicable, details of whether and how the clustering by care provides or centers was addressed

7a-i) Describe whether and how expected attrition was taken into account when calculating the sample size

Describe whether and how expected attrition was taken into account when calculating the sample size.

1

2

3

4

5

subitem not at all important

essential

Does your paper address subitem 7a-i?

Copy and paste relevant sections from manuscript title (include quotes in quotation marks "like this" to indicate direct quotes from your manuscript), or elaborate on this item by providing additional information not in the ms, or briefly explain why the item is not applicable/relevant for your study

"We used a two-sided test and assumed a binomial distribution, a 95% confidence interval, 80% power, and an absolute change of 10% in IDM. Therefore, 776 Turkish- and 794 Moroccan-Dutch women (in total; both the control and intervention group) were needed.

This absolute change of 10% in IDM was based on a previously reported increase of 11% in IDM regarding prenatal screening among pregnant ethnic minority women in the

Netherlands due to a developed CSEV [8]."

7b) When applicable, explanation of any interim analyses and stopping guidelines

Does your paper address CONSORT subitem 7b? *

Copy and paste relevant sections from the manuscript (include quotes in quotation marks "like this" to indicate direct quotes from your manuscript), or elaborate on this item by providing additional information not in the ms, or briefly explain why the item is not applicable/relevant for your study

There were no interim analyses and stopping guidelines.

8a) Method used to generate the random allocation sequence

NPT: When applicable, how care providers were allocated to each trial group

Does your paper address CONSORT subitem 8a? *

Copy and paste relevant sections from the manuscript (include quotes in quotation marks "like this" to indicate direct quotes from your manuscript), or elaborate on this item by providing additional information not in the ms, or briefly explain why the item is not applicable/relevant for your study

"RDS starts with a convenience, ideally diverse, sample of members of the population, which are called seeds [11]. Seeds are asked to complete a questionnaire and to recruit a number of their peers to complete the same questionnaire. Successfully recruited peers are then also asked to recruit a number of peers. This recruitment was continued until the calculated sample size was reached. Unique tokens were used to follow who recruited whom and draw recruitment trees. Each new respondent was randomly assigned to either the control or

intervention condition (i.e. individual level randomization) (see Figure 1)."

8b) Type of randomisation; details of any restriction (such as blocking and block size)

Does your paper address CONSORT subitem 8b? *

Copy and paste relevant sections from the manuscript (include quotes in quotation marks "like this" to indicate direct quotes from your manuscript), or elaborate on this item by providing additional information not in the ms, or briefly explain why the item is not applicable/relevant for your study

"RDS starts with a convenience, ideally diverse, sample of members of the population, which are called seeds [11]. Seeds are asked to complete a questionnaire and to recruit a number of their peers to complete the same questionnaire. Successfully recruited peers are then also asked to recruit a number of peers. This recruitment was continued until the calculated sample size was reached. Unique tokens were used to follow who recruited whom and draw recruitment trees. Each new respondent was randomly assigned to either the control or

intervention condition (i.e. individual level randomization) (see Figure 1)."

9) Mechanism used to implement the random allocation sequence (such as sequentially numbered containers), describing any steps taken to conceal the sequence until interventions were assigned

Does your paper address CONSORT subitem 9? *

Copy and paste relevant sections from the manuscript (include quotes in quotation marks "like this" to indicate direct quotes from your manuscript), or elaborate on this item by providing additional information not in the ms, or briefly explain why the item is not applicable/relevant for your study

"RDS starts with a convenience, ideally diverse, sample of members of the population, which are called seeds [11]. Seeds are asked to complete a questionnaire and to recruit a number of their peers to complete the same questionnaire. Successfully recruited peers are then also asked to recruit a number of peers. This recruitment was continued until the calculated sample size was reached. Unique tokens were used to follow who recruited whom and draw recruitment trees. Each new respondent was randomly assigned to either the control or

intervention condition (i.e. individual level randomization) (see Figure 1)."

10) Who generated the random allocation sequence, who enrolled participants, and who assigned participants to interventions

Does your paper address CONSORT subitem 10? *

Copy and paste relevant sections from the manuscript (include quotes in quotation marks "like this" to indicate direct quotes from your manuscript), or elaborate on this item by providing additional information not in the ms, or briefly explain why the item is not applicable/relevant for your study

"RDS starts with a convenience, ideally diverse, sample of members of the population, which are called seeds [11]. Seeds are asked to complete a questionnaire and to recruit a number of their peers to complete the same questionnaire. Successfully recruited peers are then also asked to recruit a number of peers. This recruitment was continued until the calculated sample size was reached. Unique tokens were used to follow who recruited whom and draw recruitment trees. Each new respondent was randomly assigned to either the control or

intervention condition (i.e. individual level randomization) (see Figure 1)."

11a) If done, who was blinded after assignment to interventions (for example, participants, care providers, those assessing outcomes) and how

NPT: Whether or not administering co-interventions were blinded to group assignment

11a-i) Specify who was blinded, and who wasn’t

Specify who was blinded, and who wasn’t. Usually, in web-based trials it is not possible to blind the participants [1, 3] (this should be clearly acknowledged), but it may be possible to blind outcome assessors, those doing data analysis or those administering co-interventions (if any).

1

2

3

4

5

subitem not at all important

essential

Does your paper address subitem 11a-i? *

Copy and paste relevant sections from the manuscript (include quotes in quotation marks "like this" to indicate direct quotes from your manuscript), or elaborate on this item by providing additional information not in the ms, or briefly explain why the item is not applicable/relevant for your study

"Respondents were informed about the study (but did not know there was a control and an

intervention group), and asked to give their digital informed consent."

11a-ii) Discuss e.g., whether participants knew which intervention was the “intervention of interest” and which one was the “comparator”

Informed consent procedures (4a-ii) can create biases and certain expectations - discuss e.g., whether participants knew which intervention was the “intervention of interest” and which one was the “comparator”.

1

2

3

4

5

subitem not at all important

essential

Does your paper address subitem 11a-ii?

Copy and paste relevant sections from the manuscript (include quotes in quotation marks "like this" to indicate direct quotes from your manuscript), or elaborate on this item by providing additional information not in the ms, or briefly explain why the item is not applicable/relevant for your study

"Respondents were informed about the study (but did not know there was a control and an

intervention group), and asked to give their digital informed consent."

11b) If relevant, description of the similarity of interventions

(this item is usually not relevant for ehealth trials as it refers to similarity of a placebo or sham intervention to a active medication/intervention)

Does your paper address CONSORT subitem 11b? *

Copy and paste relevant sections from the manuscript (include quotes in quotation marks "like this" to indicate direct quotes from your manuscript), or elaborate on this item by providing additional information not in the ms, or briefly explain why the item is not applicable/relevant for your study

"The control group was asked to read the information brochure regarding the screening program that is currently sent with the screening invitation. The intervention group was

asked to read the same brochure and watch the CSEV."

12a) Statistical methods used to compare groups for primary and secondary outcomes

NPT: When applicable, details of whether and how the clustering by care providers or centers was

addressed

Does your paper address CONSORT subitem 12a? *

Copy and paste relevant sections from the manuscript (include quotes in quotation marks "like this" to indicate direct quotes from your manuscript), or elaborate on this item by providing additional information not in the ms, or briefly explain why the item is not applicable/relevant for your study

"To analyze the potential additional effect of the CSEV compared to that of the brochure only, we conducted intention-to-treat analyses [16]. We assessed the differences in knowledge (or awareness in case of self-sampling), attitude (or perceptions in case of self- sampling), intention, and IDM (only for clinician-based sampling) between the control and intervention group after the control or intervention condition using Chi-square tests or

Fisher’s exact tests."

12a-i) Imputation techniques to deal with attrition / missing values

Imputation techniques to deal with attrition / missing values: Not all participants will use the intervention/comparator as intended and attrition is typically high in ehealth trials. Specify how participants who did not use the application or dropped out from the trial were treated in the statistical analysis (a complete case analysis is strongly discouraged, and simple imputation techniques such as LOCF may also be problematic [4]).

1

2

3

4

5

subitem not at all important

essential

Does your paper address subitem 12a-i? *

Copy and paste relevant sections from the manuscript (include quotes in quotation marks "like this" to indicate direct quotes from your manuscript), or elaborate on this item by providing additional information not in the ms, or briefly explain why the item is not applicable/relevant for your study

"To analyze the potential additional effect of the CSEV compared to that of the brochure only, we conducted intention-to-treat analyses [16]. We assessed the differences in knowledge (or awareness in case of self-sampling), attitude (or perceptions in case of self- sampling), intention, and IDM (only for clinician-based sampling) between the control and intervention group after the control or intervention condition using Chi-square tests or

Fisher’s exact tests."

12b) Methods for additional analyses, such as subgroup analyses and adjusted analyses

x26-ii) Outline informed consent procedures

Outline informed consent procedures e.g., if consent was obtained offline or online (how? Checkbox, etc.?), and what information was provided (see 4a-ii). See [6] for some items to be included in informed consent documents.

1

2

3

4

5

subitem not at all important

essential

Does your paper address CONSORT subitem 12b? *

Copy and paste relevant sections from the manuscript (include quotes in quotation marks "like this" to indicate direct quotes from your manuscript), or elaborate on this item by providing additional information not in the ms, or briefly explain why the item is not applicable/relevant for your study

"As a post-hoc analysis, we explored the open-field comments stated by respondents at the end of our questionnaire, to explain differences found between Turkish- and Moroccan-

Dutch women."

X26) REB/IRB Approval and Ethical Considerations [recommended as subheading under "Methods"] (not a CONSORT item)

X26-i) Comment on ethics committee approval

1

2

3

4

5

subitem not at all important

essential

Does your paper address subitem X26-i?

Copy and paste relevant sections from the manuscript (include quotes in quotation marks "like this" to indicate direct quotes from your manuscript), or elaborate on this item by providing additional information not in the ms, or briefly explain why the item is not applicable/relevant for your study

"After the Medical Ethics Review Committee of the University Medical Centre Utrecht confirmed that the Medical Research Involving Human Subjects Act does not apply to this

study (nr: 20/105), we registered the trial at the Netherlands Trial Register (nr: NL8453)."

Does your paper address subitem X26-ii?

Copy and paste relevant sections from the manuscript (include quotes in quotation marks "like this" to indicate direct quotes from your manuscript), or elaborate on this item by providing additional information not in the ms, or briefly explain why the item is not applicable/relevant for your study

"Respondents were informed about the study (but did not know there was a control and an

intervention group), and asked to give their digital informed consent."

X26-iii) Safety and security procedures

Safety and security procedures, incl. privacy considerations, and any steps taken to reduce the likelihood or detection of harm (e.g., education and training, availability of a hotline)

1

2

3

4

5

subitem not at all important

essential

Does your paper address subitem X26-iii?

Copy and paste relevant sections from the manuscript (include quotes in quotation marks "like this" to indicate direct quotes from your manuscript), or elaborate on this item by providing additional information not in the ms, or briefly explain why the item is not applicable/relevant for your study

Jouw antwoord

RESULTS

13a) For each group, the numbers of participants who were randomly assigned, received intended treatment, and were analysed for the primary outcome

NPT: The number of care providers or centers performing the intervention in each group and the

number of patients treated by each care provider in each center

Does your paper address CONSORT subitem 13a? *

Copy and paste relevant sections from the manuscript (include quotes in quotation marks "like this" to indicate direct quotes from your manuscript), or elaborate on this item by providing additional information not in the ms, or briefly explain why the item is not applicable/relevant for your study

"Of the 2948 respondents that started the questionnaire, 1931 (65.5%) completed it. After excluding 367 respondents (19.0%), 686 Turkish- and 878 Moroccan-Dutch women were included for analysis: 793 in the control group (350 Turkish and 443 Moroccan), and 771 in

the intervention group (336 Turkish and 435 Moroccan)."

13b) For each group, losses and exclusions after randomisation, together with reasons

Does your paper address CONSORT subitem 13b? (NOTE: Preferably, this is shown in a CONSORT flow diagram) *

Copy and paste relevant sections from the manuscript (include quotes in quotation marks "like this" to indicate direct quotes from your manuscript), or elaborate on this item by providing additional information not in the ms, or briefly explain why the item is not applicable/relevant for your study

This is shown in a CONSORT flow diagram (see Figure 2).

13b-i) Attrition diagram

Strongly recommended: An attrition diagram (e.g., proportion of participants still logging in or using the intervention/comparator in each group plotted over time, similar to a survival curve) or other figures or tables demonstrating usage/dose/engagement.

1

2

3

4

5

subitem not at all important

essential

Does your paper address subitem 13b-i?

Copy and paste relevant sections from the manuscript or cite the figure number if applicable (include quotes in quotation marks "like this" to indicate direct quotes from your manuscript), or elaborate on this item by providing additional information not in the ms, or briefly explain why the item is not applicable/relevant for your study

Jouw antwoord

14a) Dates defining the periods of recruitment and follow-up

Does your paper address CONSORT subitem 14a? *

Copy and paste relevant sections from the manuscript (include quotes in quotation marks "like this" to indicate direct quotes from your manuscript), or elaborate on this item by providing additional information not in the ms, or briefly explain why the item is not applicable/relevant for your study

"Between 23 November 2020 and 6 August 2021, a randomized intervention study was

conducted with a control and an intervention group."

14a-i) Indicate if critical “secular events” fell into the study period

Indicate if critical “secular events” fell into the study period, e.g., significant changes in Internet resources available or “changes in computer hardware or Internet delivery resources”

1

2

3

4

5

subitem not at all important

essential

Does your paper address subitem 14a-i?

Copy and paste relevant sections from the manuscript (include quotes in quotation marks "like this" to indicate direct quotes from your manuscript), or elaborate on this item by providing additional information not in the ms, or briefly explain why the item is not applicable/relevant for your study

Jouw antwoord

14b) Why the trial ended or was stopped (early)

Does your paper address CONSORT subitem 14b? *

Copy and paste relevant sections from the manuscript (include quotes in quotation marks "like this" to indicate direct quotes from your manuscript), or elaborate on this item by providing additional information not in the ms, or briefly explain why the item is not applicable/relevant for your study

"RDS starts with a convenience, ideally diverse, sample of members of the population, which are called seeds [11]. Seeds are asked to complete a questionnaire and to recruit a number of their peers to complete the same questionnaire. Successfully recruited peers are then also asked to recruit a number of peers. This recruitment was continued until the calculated

sample size was reached."

15) A table showing baseline demographic and clinical characteristics for each group

NPT: When applicable, a description of care providers (case volume, qualification, expertise, etc.) and

centers (volume) in each group

Does your paper address CONSORT subitem 15? *

Copy and paste relevant sections from the manuscript (include quotes in quotation marks "like this" to indicate direct quotes from your manuscript), or elaborate on this item by providing additional information not in the ms, or briefly explain why the item is not applicable/relevant for your study

This is shown in Table 1. Sample characteristics of Turkish- and Moroccan-Dutch

respondents.

15-i) Report demographics associated with digital divide issues

In ehealth trials it is particularly important to report demographics associated with digital divide issues, such as age, education, gender, social-economic status, computer/Internet/ehealth literacy of the participants, if known.

1

2

3

4

5

subitem not at all important

essential

Does your paper address subitem 15-i? *

Copy and paste relevant sections from the manuscript (include quotes in quotation marks "like this" to indicate direct quotes from your manuscript), or elaborate on this item by providing additional information not in the ms, or briefly explain why the item is not applicable/relevant for your study

This is shown in Table 1. Sample characteristics of Turkish- and Moroccan-Dutch

respondents.

16) For each group, number of participants (denominator) included in each analysis and whether the analysis was by original assigned groups

16-i) Report multiple “denominators” and provide definitions

Report multiple “denominators” and provide definitions: Report N’s (and effect sizes) “across a range of study participation [and use] thresholds” [1], e.g., N exposed, N consented, N used more than x times, N used more than y weeks, N participants “used” the intervention/comparator at specific pre-defined time points of interest (in absolute and relative numbers per group). Always clearly define “use” of the intervention.

1

2

3

4

5

subitem not at all important

essential

Does your paper address subitem 16-i? *

Copy and paste relevant sections from the manuscript (include quotes in quotation marks "like this" to indicate direct quotes from your manuscript), or elaborate on this item by providing additional information not in the ms, or briefly explain why the item is not applicable/relevant for your study

This is shown in Table 2 and Table 3.

16-ii) Primary analysis should be intent-to-treat

Primary analysis should be intent-to-treat, secondary analyses could include comparing only “users”, with the appropriate caveats that this is no longer a randomized sample (see 18-i).

1

2

3

4

5

subitem not at all important

essential

Does your paper address subitem 16-ii?

Copy and paste relevant sections from the manuscript (include quotes in quotation marks "like this" to indicate direct quotes from your manuscript), or elaborate on this item by providing additional information not in the ms, or briefly explain why the item is not applicable/relevant for your study

"To analyze the potential additional effect of the CSEV compared to that of the brochure

only, we conducted intention-to-treat analyses [16]."

17a) For each primary and secondary outcome, results for each group, and the estimated effect size and its precision (such as 95% confidence interval)

Does your paper address CONSORT subitem 17a? *

Copy and paste relevant sections from the manuscript (include quotes in quotation marks "like this" to indicate direct quotes from your manuscript), or elaborate on this item by providing additional information not in the ms, or briefly explain why the item is not applicable/relevant for your study

This is shown in Table 2 and Table 3.

17a-i) Presentation of process outcomes such as metrics of use and intensity of use

In addition to primary/secondary (clinical) outcomes, the presentation of process outcomes such as metrics of use and intensity of use (dose, exposure) and their operational definitions is critical. This does not only refer to metrics of attrition (13-b) (often a binary variable), but also to more continuous exposure metrics such as “average session length”. These must be accompanied by a technical description how a metric like a “session” is defined (e.g., timeout after idle time) [1] (report under item 6a).

1

2

3

4

5

subitem not at all important

essential

Does your paper address subitem 17a-i?

Copy and paste relevant sections from the manuscript (include quotes in quotation marks "like this" to indicate direct quotes from your manuscript), or elaborate on this item by providing additional information not in the ms, or briefly explain why the item is not applicable/relevant for your study

Jouw antwoord

17b) For binary outcomes, presentation of both absolute and relative effect sizes is recommended

Does your paper address CONSORT subitem 17b? *

Copy and paste relevant sections from the manuscript (include quotes in quotation marks "like this" to indicate direct quotes from your manuscript), or elaborate on this item by providing additional information not in the ms, or briefly explain why the item is not applicable/relevant for your study

This is shown in Table 2 and Table 3.

18) Results of any other analyses performed, including subgroup analyses and adjusted analyses, distinguishing pre-specified from exploratory

Does your paper address CONSORT subitem 18? *

Copy and paste relevant sections from the manuscript (include quotes in quotation marks "like this" to indicate direct quotes from your manuscript), or elaborate on this item by providing additional information not in the ms, or briefly explain why the item is not applicable/relevant for your study

No any other analyses have been reported.

18-i) Subgroup analysis of comparing only users

A subgroup analysis of comparing only users is not uncommon in ehealth trials, but if done, it must be stressed that this is a self-selected sample and no longer an unbiased sample from a randomized trial (see 16-iii).

1

2

3

4

5

subitem not at all important

essential

19) All important harms or unintended effects in each group

(for specific guidance see CONSORT for harms)

Does your paper address CONSORT subitem 19? *

Copy and paste relevant sections from the manuscript (include quotes in quotation marks "like this" to indicate direct quotes from your manuscript), or elaborate on this item by providing additional information not in the ms, or briefly explain why the item is not applicable/relevant for your study

No harms have been indicated/reported. As for unintended effects: "The brochure has a significant positive influence on IDM, while the CSEV has an added effect on the attitude towards CC screening in especially Moroccan-Dutch women. These women had more often a positive attitude towards CC screening compared to the control group with only the brochure. This was especially the case among women who had never participated in CC screening. Based on the open-field comments of Turkish-Dutch respondents, we think we can explain why this effect was not visible in this group. It appeared that some of the Turkish-Dutch respondents were offended by the fact that in the Turkish video the actress

who played the negative screening attitude was wearing a headscarf."

19-i) Include privacy breaches, technical problems

Include privacy breaches, technical problems. This does not only include physical “harm” to participants, but also incidents such as perceived or real privacy breaches [1], technical problems, and other unexpected/unintended incidents. “Unintended effects” also includes unintended positive effects [2].

1

2

3

4

5

subitem not at all important

essential

Does your paper address subitem 19-i?

Copy and paste relevant sections from the manuscript (include quotes in quotation marks "like this" to indicate direct quotes from your manuscript), or elaborate on this item by providing additional information not in the ms, or briefly explain why the item is not applicable/relevant for your study

Jouw antwoord

19-ii) Include qualitative feedback from participants or observations from staff/researchers

Include qualitative feedback from participants or observations from staff/researchers, if available, on strengths and shortcomings of the application, especially if they point to unintended/unexpected effects or uses. This includes (if available) reasons for why people did or did not use the application as intended by the developers.

1

2

3

4

5

subitem not at all important

essential

Does your paper address subitem 19-ii?

Copy and paste relevant sections from the manuscript (include quotes in quotation marks "like this" to indicate direct quotes from your manuscript), or elaborate on this item by providing additional information not in the ms, or briefly explain why the item is not applicable/relevant for your study

Jouw antwoord

DISCUSSION

22) Interpretation consistent with results, balancing benefits and harms, and considering other relevant evidence

NPT: In addition, take into account the choice of the comparator, lack of or partial blinding, and unequal

expertise of care providers or centers in each group

22-i) Restate study questions and summarize the answers suggested by the data, starting with primary outcomes and process outcomes (use)

Restate study questions and summarize the answers suggested by the data, starting with primary outcomes and process outcomes (use).

1

2

3

4

5

subitem not at all important

essential

# Does your paper address subitem 22-i? *

Copy and paste relevant sections from the manuscript (include quotes in quotation marks "like this" to indicate direct quotes from your manuscript), or elaborate on this item by providing additional information not in the ms, or briefly explain why the item is not applicable/relevant for your study

"This study evaluated the effect of a Culturally Sensitive Educational Video (CSEV) on knowledge, attitude, intention, and informed decision-making (IDM) regarding CC screening among Turkish- and Moroccan-Dutch women aged 30-60 years. The CSEV was watched far more often than the brochure when both were offered, and the intervention group who watched the video also studied the brochure more often than the control group. The brochure has a significant positive influence on IDM, while the CSEV has an added effect on the attitude towards CC screening in especially Moroccan-Dutch women. These women had more often a positive attitude towards CC screening compared to the control group with only the brochure. This was especially the case among women who had never participated in CC screening. Based on the open-field comments of Turkish-Dutch respondents, we think we can explain why this effect was not visible in this group. It appeared that some of the Turkish-Dutch respondents were offended by the fact that in the Turkish video the actress who played the negative screening attitude was wearing a headscarf."

22-ii) Highlight unanswered new questions, suggest future research

Highlight unanswered new questions, suggest future research.

1

2

3

4

5

subitem not at all important

essential

Does your paper address subitem 22-ii?

Copy and paste relevant sections from the manuscript (include quotes in quotation marks "like this" to indicate direct quotes from your manuscript), or elaborate on this item by providing additional information not in the ms, or briefly explain why the item is not applicable/relevant for your study

Jouw antwoord

20) Trial limitations, addressing sources of potential bias, imprecision, and, if relevant, multiplicity of analyses

20-i) Typical limitations in ehealth trials

Typical limitations in ehealth trials: Participants in ehealth trials are rarely blinded. Ehealth trials often look at a multiplicity of outcomes, increasing risk for a Type I error. Discuss biases due to non-use of the intervention/usability issues, biases through informed consent procedures, unexpected events.

1

2

3

4

5

subitem not at all important

essential

# Does your paper address subitem 20-i? *

Copy and paste relevant sections from the manuscript (include quotes in quotation marks "like this" to indicate direct quotes from your manuscript), or elaborate on this item by providing additional information not in the ms, or briefly explain why the item is not applicable/relevant for your study

"However, a number of limitations should also be addressed.

First, due to the online delivery, we sampled more 30-39 year old’s, those of second generation, and highly educated Turkish- and Moroccan-Dutch women, compared to the national dataset of 2020 of Statistics Netherlands [17-19]. Still, the two randomized groups were comparable, and 12% and 8% of the Turkish- and Moroccan-Dutch respondents reported no official education or completed primary school, respectively. Also, regarding previous CC screening participation, we did found similar rates of at least one participation in CC screening of 60% and 64% of Turkish- and Moroccan-Dutch women versus 64% and 53%, respectively, in previous reports [6].

Second, the time elapsed between the previous screening invitation and the questionnaire administration, which varied largely among our respondents, might have impacted the experienced relevance of the decision-making questions and the previously existing knowledge. However, this heterogeneity is likely to play a similar role (if it does at all) in both the control and intervention group because of the performed randomization.

Third, women participating in our study might have been different from those not participating. They could, for example, be more interested in CC screening as a topic, and thus be more informed about the screening than non-participating women. Nevertheless, since we used incentives for successful peer recruitment, this might also have been the reason for some respondents to participate in the study, rather than being interested in CC screening. In addition, this possible selection bias is likely to be present in both the control and intervention group and should not affect the evaluation of the CSEV.

Fourth, our knowledge construct contained only some facts about CC screening (i.e. process after a negative/positive test result and the possibility of false-positive test results). Although these have been carefully selected, they do not cover the entire spectrum of decision-relevant information (e.g. hrHPV as the causative agent of CC and its transmission route) and could only indicate some deficits. Because of the use of RDS, and thus requesting women to successfully recruit others, we aimed to burden the respondents as less as possible and, therefore, keep the questionnaire as short as possible.

Finally, we based the content of the CSEVs on our earlier conducted focus groups among offline-recruited Turkish- and Moroccan-Dutch women [5]. Because of the measures for the ongoing COVID-19 pandemic (e.g. nationwide lockdowns), we were unable to approach potential respondents face-to-face and recruit them offline. The respondents, as well, were unable to recruit peers offline unless they were household members. This resulted in an online-only, relatively young, mostly second-generation sample of Turkish- and Moroccan- Dutch women. It would be highly relevant to evaluate the CSEVs in an offline setting, comparable with our previous study [5]. We believe the CSEVs could affect IDM (greater) in such a setting, for which the CSEVs were tailored during the development process."

21) Generalisability (external validity, applicability) of the trial findings

NPT: External validity of the trial findings according to the intervention, comparators, patients, and care providers or centers involved in the trial

21-i) Generalizability to other populations

Generalizability to other populations: In particular, discuss generalizability to a general Internet population, outside of a RCT setting, and general patient population, including applicability of the study results for other organizations

1

2

3

4

5

subitem not at all important

essential

Does your paper address subitem 21-i?

Copy and paste relevant sections from the manuscript (include quotes in quotation marks "like this" to indicate direct quotes from your manuscript), or elaborate on this item by providing additional information not in the ms, or briefly explain why the item is not applicable/relevant for your study

Jouw antwoord

21-ii) Discuss if there were elements in the RCT that would be different in a routine application setting

Discuss if there were elements in the RCT that would be different in a routine application setting (e.g., prompts/reminders, more human involvement, training sessions or other co-interventions) and what impact the omission of these elements could have on use, adoption, or outcomes if the intervention is applied outside of a RCT setting.

1

2

3

4

5

subitem not at all important

essential

Does your paper address subitem 21-ii?

Copy and paste relevant sections from the manuscript (include quotes in quotation marks "like this" to indicate direct quotes from your manuscript), or elaborate on this item by providing additional information not in the ms, or briefly explain why the item is not applicable/relevant for your study

Jouw antwoord

OTHER INFORMATION

23) Registration number and name of trial registry

Does your paper address CONSORT subitem 23? *

Copy and paste relevant sections from the manuscript (include quotes in quotation marks "like this" to indicate direct quotes from your manuscript), or elaborate on this item by providing additional information not in the ms, or briefly explain why the item is not applicable/relevant for your study

"After the Medical Ethics Review Committee of the University Medical Centre Utrecht confirmed that the Medical Research Involving Human Subjects Act does not apply to this

study (nr: 20/105), we registered the trial at the Netherlands Trial Register (nr: NL8453)."

24) Where the full trial protocol can be accessed, if available

Does your paper address CONSORT subitem 24? *

Cite a Multimedia Appendix, other reference, or copy and paste relevant sections from the manuscript (include quotes in quotation marks "like this" to indicate direct quotes from your manuscript), or elaborate on this item by providing additional information not in the ms, or briefly explain why the item is not applicable/relevant for your study

The full trial protocol is not available.

25) Sources of funding and other support (such as supply of drugs), role of funders

Does your paper address CONSORT subitem 25? *

Copy and paste relevant sections from the manuscript (include quotes in quotation marks "like this" to indicate direct quotes from your manuscript), or elaborate on this item by providing additional information not in the ms, or briefly explain why the item is not applicable/relevant for your study

"This study was part of a larger research project called the FEMININE study, which was

funded by the Netherlands Organisation for Health Research and Development (nr: 531002030)."

X27) Conflicts of Interest (not a CONSORT item)

X27-i) State the relation of the study team towards the system being evaluated

In addition to the usual declaration of interests (financial or otherwise), also state the relation of the study team towards the system being evaluated, i.e., state if the authors/evaluators are distinct from or identical with the developers/sponsors of the intervention.

1

2

3

4

5

subitem not at all important

essential

Does your paper address subitem X27-i?

Copy and paste relevant sections from the manuscript (include quotes in quotation marks "like this" to indicate direct quotes from your manuscript), or elaborate on this item by providing additional information not in the ms, or briefly explain why the item is not applicable/relevant for your study

Jouw antwoord

About the CONSORT EHEALTH checklist

As a result of using this checklist, did you make changes in your manuscript? *

yes, major changes

yes, minor changes no

What were the most important changes you made as a result of using this

checklist?

Jouw antwoord

How much time did you spend on going through the checklist INCLUDING

making changes in your manuscript *

I spend approximately two hours going through the checklist (including making changes).

As a result of using this checklist, do you think your manuscript has improved? *

yes no

Anders:

Would you like to become involved in the CONSORT EHEALTH group?

This would involve for example becoming involved in participating in a workshop and writing an "Explanation and Elaboration" document

yes no

Anders:

Selectie wissen

Any other comments or questions on CONSORT EHEALTH

Jouw antwoord

STOP - Save this form as PDF before you click submit

To generate a record that you filled in this form, we recommend to generate a PDF of this page (on a Mac, simply select "print" and then select "print as PDF") before you submit it.

When you submit your (revised) paper to JMIR, please upload the PDF as supplementary file.

Don't worry if some text in the textboxes is cut off, as we still have the complete information in our database. Thank you!

Final step: Click submit !

Click submit so we have your answers in our database!

Verzenden Formulier wissen

Verzend nooit wachtwoorden via Google Formulieren.

Deze content is niet gemaakt of goedgekeurd door Google. M [isbruik rapporteren](https://docs.google.com/forms/u/0/d/e/1FAIpQLSfZBSUp1bwOc_OimqcS64RdfIAFvmrTSkZQL2-3O8O9hrL5Sw/reportabuse?hl=en_US&source=https%3A//docs.google.com/forms/d/e/1FAIpQLSfZBSUp1bwOc_OimqcS64RdfIAFvmrTSkZQL2-3O8O9hrL5Sw/viewform%3Fhl%3Den_US%26hl%3Den_US%26formkey%3DdGlKd2Z2Q1lNSGQ0THl1azM5MS1aWWc6MA%26rm%3Dfull) - [S ervicevoorwaarden](https://policies.google.com/terms) - [Privacybeleid](https://policies.google.com/privacy)

[Formulieren](https://www.google.com/forms/about/?utm_source=product&utm_medium=forms_logo&utm_campaign=forms)
